# β-catenin initiates peritoneal fibrosis by triggering mitochondrial fission-mediated mesothelial cell senescence fate transition

**DOI:** 10.1186/s40779-025-00669-1

**Published:** 2025-12-01

**Authors:** Xiao-Xu Wang, Wei-Jie Zhong, Jie-Mei Li, Di Wang, Shuang-Qin Chen, Jin-Hua Miao, Wei-Wei Shen, Xiao-Long Li, Jie-Wu Huang, Shan Zhou, Cheng Wang, Jun Ai, Li-Li Zhou

**Affiliations:** 1 Division of Nephrology, Nanfang Hospital, Southern Medical University, Guangzhou, 510515 China; 2 State Key Laboratory of Multi-Organ Injury Prevention and Treatment, Nanfang Hospital, Southern Medical University, Guangzhou, 510515 China; 3 National Clinical Research Center for Kidney Disease, Nanfang Hospital, Southern Medical University, Guangzhou, 510515 China; 4 Guangdong Provincial Institute of Nephrology, Nanfang Hospital, Southern Medical University, Guangzhou, 510515 China; 5 Guangdong Provincial Key Laboratory of Renal Failure Research, Nanfang Hospital, Southern Medical University, Guangzhou, 510515 China; 6 Division of Nephrology, Department of Medicine, the Fifth Affiliated Hospital of Sun Yat-Sen University, Zhuhai, 519000 Guangdong China

**Keywords:** β-catenin, Mesothelial cell, Senescence, Fibroblast, Peritoneal fibrosis, Peritoneal dialysis (PD)

## Abstract

**Background:**

Peritoneal fibrosis represents a major clinical challenge for end-stage renal disease (ESRD) patients when they are undergoing peritoneal dialysis (PD). Single-cell RNA sequencing identified that peritoneal mesothelial cells undergo a senescence fate transition in long-term PD patients. Whereas the existence of mesothelial cell senescence and the underlying mechanisms should be thoroughly explored.

**Methods:**

To further investigate mesothelial cell senescence, we utilized a clinical cohort comprising dialysate effluents from PD patients and peritoneal biopsy specimens, peritoneal dialysis fluid (PDF)-induced mouse models, and cultured primary mesothelial cells. Single-cell RNA sequencing, transcriptome sequencing, immunofluorescence, Western blotting, and other analyses were administered. To validate the critical role of β-catenin in mesothelial cell senescence, β-catenin knockout mice were employed. Additionally, the senolytic drugs dasatinib plus quercetin were administered to PDF mice to assess the key role of mesothelial cell senescence in peritoneal fibrosis.

**Results:**

Single-cell RNA sequencing demonstrated that mesothelial cells derived from long-term PD patients are major trend to senescence fate. Moreover, β-catenin signaling was significantly upregulated, as well as transforming growth factor-β (TGF-β) pathways. We observed that senescent mesothelial cells were highly increased in both dialysate effluent and peritoneal biopsies of long-term PD patients. In dialysate effluent, matrix metalloproteinase-7 (MMP-7), an indicator of downstream targets of β-catenin, was positively correlated with TGF-β1. Both biomarkers were also positively associated with PD duration. Mechanistically, we found that β-catenin promotes dynamin-related protein 1 (Drp1) expression, a key mediator of mitochondrial fission, thereby inducing mesothelial cell senescence. Then, TGF-β1 was secreted to activate the Smad signaling pathway in fibroblasts, leading to myofibroblast activation and subsequent peritoneal fibrosis. Notably, administration of senolytic drugs, dasatinib plus quercetin, significantly alleviated peritoneal fibrosis regardless of treatment timing.

**Conclusion:**

Targeting β-catenin signaling and mesothelial cell senescence may represent potential therapeutic interventions for preventing peritoneal fibrosis.

**Supplementary Information:**

The online version contains supplementary material available at 10.1186/s40779-025-00669-1.

## Background

Peritoneal dialysis (PD) is a kind of therapy for the replacement of the kidney, which is widely used in patients with end-stage renal disease (ESRD). PD has unique benefits of convenience and superiority in protecting residual kidney function [1, 2]. However, exposure to the bioincompatible peritoneal dialysis fluid (PDF) for a long time results in peritoneal fibrosis, which not only leads to PD failure but also triggers the onset of encapsulating peritoneal sclerosis with a high mortality rate [3, 4].

Mesothelial cells, the first barrier in contact with PDF, possess secretion and substance exchange functions [5]. In this process, they are vulnerable when immersed in the high-glucose microenvironment of PDF. Numerous reports show that mesothelial cells could undergo mesothelial-mesenchymal transition (MMT), mitochondrial injury, and apoptosis, all of which contribute to peritoneal fibrosis [6–8]. Moreover, mesothelial cells and fibroblasts communicate with each other, which is critical for peritoneal fibrosis [9, 10]. However, the underlying mechanisms of mesothelial cell injury and their communication with fibroblasts remain poorly understood.

Cell senescence presents cell cycle arrest perpetually, accompanied by the secretion of senescence-associated secretory phenotype (SASP) molecules [11]. Senescence is likely to occur in cells with high metabolic needs [12]. Mesothelial cells frequently engage in substance exchange, indicating their high energy requirements. In addition, in PD patients, mesothelial cells are chronically exposed to high-glucose PDF, making them prone to senescence [13]. Indeed, recent studies have demonstrated that mesothelial cells exhibit a senescence-like phenotype in PDF-treated mouse models [14, 15]. In addition, several previous reports have identified classical hallmarks of cellular senescence in peritoneal mesothelial cells, such as a reduced mitotic index, hypertrophic morphology, multinucleation, and DNA damage [13, 16]. Collectively, these findings indicate that mesothelial cell senescence occurs during PD treatment. However, direct evidence of mesothelial cell senescence in clinical samples remains limited. Moreover, a comprehensive trajectory analysis of cell senescence fate transitions in mesothelial cells has not yet been conducted. The underlying mechanisms driving mesothelial cell senescence also require further elucidation.

Mitochondria are vital organelles for regulating energy metabolism and cell homeostasis in all eukaryotic cells, including peritoneal mesothelial cells. Mitochondrial damage highly induces mesothelial cell injury through the induction of reactive oxygen species (ROS) [17, 18]. Notably, as dynamic organelles, mitochondria are frequently undergoing fusion and fission. Excessive mitochondrial fission has been observed in mesothelial cells under long-term PD [7, 19]. Dynamin-related protein 1 (Drp1), an essential factor that modulates mitochondrial fission, is highly involved in ROS production, adenosine triphosphate (ATP) depletion, mitochondrial DNA (mtDNA) damage, and cellular senescence [20–22]. However, the underlying mechanisms of Drp1 regulation and mitochondrial fission in peritoneal mesothelial cells remain undefined.

β-catenin signaling participates in development and repair, which is evolutionarily conserved [23]. By binding to the transcription factors of T-cell factor (TCF) or lymphoid enhancer binding factor (LEF), β-catenin regulates various genes, including those encoding matrix metalloproteinases and components of the renin-angiotensin-aldosterone system [24]. Several inhibitors to β-catenin signaling, such as ICG-001, Klotho, and dickkopf-related protein 1, have been shown to suppress peritoneal fibrosis [25–27]. Our previous studies revealed that β-catenin highly promotes senescence in renal tubular cells and plays a role in tubular cells’ communication with interstitial fibroblasts [28, 29]. In addition, β-catenin signaling promotes Drp1-mediated mitochondrial fission in other cell types [30, 31]. However, the role of β-catenin in mesothelial cells and its association with mitochondrial dysfunction remains unclear.

Herein, this study aimed to prove the existence of cell senescence in peritoneal mesothelial cells and define the critical involvement of β-catenin in the process. The prospects of senolytic therapy in peritoneal fibrosis were also explored. This study would provide the key mechanisms for peritoneal fibrosis and propose potential intervention strategies in the future.

## Methods

### Patient samples

The clinical samples were from the inpatients at Nanfang Hospital of Southern Medical University from June 2020 to June 2024. The research was approved by the Ethics Committees for Human Research (NFEC-2019–107). PD patients, 18 – 60 years old, were enrolled; however, they would be excluded when peritonitis, malignancy, or abdominal surgery (except catheter implantation or removal surgery) in the recent 3 months were present.

Peritoneum samples were collected from the parietal peritoneum of PD patients receiving implantation (predialysis PD patients, *n* = 5) or removal (PD vintage > 30 months, *n* = 5) of a catheter, following informed consent approval. Paraffin or OCT was used to embed samples. Enrolled patients’ clinical features are listed in Additional file 1: Tables S1.

Primary human mesothelial cells were obtained and isolated from dialysate effluent of PD patients (short-term PD, *n* = 6, PD duration ≤ 3 months; long-term PD, *n* = 6, PD duration > 60 months). The enrolled patients’ information is presented in Additional file 1: Table S2.

Additionally, dialysate effluent was collected from 51 long-term PD patients (PD duration ranging from 3 to 12 years) during peritoneal equilibration testing. Enrolled patients’ clinical features are listed in Additional file 1: Table S3.

### Animal experiments

Animal studies (IACUC-LAC-20240513–001) were authorized by the Animal Ethics Committee of Nanfang Hospital. 15 male β-catenin^loxP/loxP^ mice, in C57BL/6 background, 8 weeks old, were cultivated from Cyagen Biosciences company (CKOCMP-12387-Ctnnb1-B6N-VA; Cyagen Biosciences, Guangzhou, China). These β-catenin^loxP/loxP^ mice were separated into 3 groups in a random manner: Saline + AAV1-ctrl (*n* = 5), PDF + AAV1-ctrl (*n* = 5), and PDF + AAV1-cre (*n* = 5). Mice received an intraperitoneal injection with AAV1-CMV-Cre-green fluorescent protein (GFP) viruses [AAV1 that carried the Cre recombinase and *GFP* gene under CMV promoter] or AAV1-control viruses (WeiZhen Biosciences, China) (1 × 10^11^ vg/g) 3 weeks ahead of the experiment. For establishing the mouse model of peritoneal fibrosis (PDF), these mice were administered with PDF (dianeal containing 4.25% glucose; BaxterHealthcare; Guangzhou; China) by intraperitoneal injection once per day, at 100 ml/kg body weight. During the experiments, all mice were alive. Six weeks later, all mice were sacrificed, and peritoneal tissue samples were harvested.

To testify to the therapeutic effects of KYA1797K (S8327, Selleck, USA), 15 male C57BL/6 mice, at the age of 8 weeks, were purchased from the Animal Center of Southern Medical University (Guangzhou, China). By random separation, they were distributed to 3 groups: Ctrl (Saline, *n* = 5), PDF (*n* = 5), and PDF + KYA1797K (*n* = 5). After dissolving in DMSO, KYA1797K was intraperitoneally injected [10 mg/(kg·d)] for 6 weeks.

For dasatinib plus quercetin (D + Q) treatment, 24 male C57BL/6 mice (8 weeks old) were purchased from the Animal Center of Southern Medical University (Guangzhou, China) and randomly separated into 4 groups as following: 1) The control mice, receiving saline injection and vehicle gavage (*n* = 6); 2) PDF mice, receiving PDF injection and vehicle gavage (*n* = 6); 3) D + Q- early treatment (ET) mice, receiving PDF injection and D + Q gavage from the beginning (*n* = 6); 4) D + Q-late treatment (LT) mice, receiving PDF injection and D + Q gavage at a late stage (*n* = 6). PDF was intraperitoneally injected [100 ml/(kg·d)]. The dosages of dasatinib and quercetin were based on a previous study [32]. Briefly, dasatinib (5 mg/kg, S1021, Selleck, USA) and quercetin (50 mg/kg, PHR1488, Sigma, USA) (D + Q) were administered into PDF mice through gavage. For ET treatment, D + Q were administered in 100 – 150 μl 10% PEG300 (S6704; Selleck, USA) for 3 consecutive days every 2 weeks (9 doses in total). For LT treatment, D + Q were administered by oral gavage at the beginning of the 5th week after PDF injection for 3 treatments (3 doses in total). At the end of 6 weeks, all animals were sacrificed. Peritoneum samples were harvested for analysis.

### Cell culture

The whole fluid of the PD drainage bag was centrifuged at 2000 rpm for 8 min, and then the cell pellets were washed with phosphate-buffered saline (PBS). After that, cells were resuspended in M199 medium (C11150500BT; Gibco, USA), incubated with 20% fetal bovine serum (FBS; A5669701; Gibco, USA), with 1% penicillin–streptomycin (C3420-0100; Viva Cell Biosciences, China) and 1% Insulin-Transferrin-Selenium (41,400–045; Gibco, USA), at 37 °C in 5% CO_2_ incubator. The next day, nonadherent cells were washed and removed. After 7–10 d, cells reached confluence. The mesothelial cells were incubated with an antibody against cytokeratin (564,709; BD Pharmingen, USA; 1:250) at 4 °C for 1 h. Ice-cold PBS, containing 2% BSA, was used to wash for 3 cycles and resuspend the cells. Flow cytometry was used to sort purified human peritoneal mesothelial cells using a BD FACSCanto™ clinical flow cytometry system instrument (BD Bioscience, USA).

Furthermore, human peritoneal mesothelial cells were determined by immunofluorescence with fluorescein-labeled Abs including antibodies against mesothelial cell markers cytokeratin (564,709; BD Pharmingen, USA; 1:50) and vimentin (21,488; Signalway antibody, USA; 1:50), endothelial cell marker CD31 (ab24590; Abcam, USA; 1:50), and leukocyte cell marker CD45 (ab243869; Abcam, USA; 1:50).

Human peritoneal mesothelial cell line, HMrSV5 cells were generously provided by Prof. Wei Chen (Sun Yat-Sen University, Guangzhou, China) [33]. Cells were cultured using DMEM/F12 medium (C11330500BT; Gibco, USA) containing 10% FBS, 1% penicillin-streptomycin and incubated in a 5% CO_2_ incubator, at 37 °C. The cultured cells in passages 2 to 4 were utilized, with a cobblestone morphology. During this period, the cells’ doubling time was nearly the same.

Primary mouse embryonic fibroblasts (MEFs) were isolated and purified from mouse embryos as previously reported [34]. The cells were cultured using DMEM (C11995500BT; Gibco, USA), with supplementation of 10% FBS and 1% penicillin-streptomycin, and incubated at 37 °C in a 5% CO_2_ atmosphere. The detailed cell treatments are available in Additional file 1: Methods.

### Cell transfection

HMrSV5 cells were transiently transfected with siRNAs or shRNA plasmids targeting Drp1, transcription factor 4 (TCF4), or p16^INK4A^ and with negative control siRNA or control-shRNA (GenPharma, China) (siDrp1 sequence: 5’-CCCUAGCUGUAAUCACUAATT-3’, siTCF4 sequence: 5’-GGAGGUACAGACAAAGAAATT-3’, p16^INK4A^-shRNA sequence: 5'-CACCAGAGGCAGUAACCA UTT-3'). The cells were subsequently transfected with a Flag-tagged β-catenin expression plasmid. Lipofectamine 8000 transfection reagent (C0533FT; Beyotime, China) was used for siRNA, shRNA, and plasmid transfection.

### Western blotting analyses

Tissue homogenate from the visceral peritoneum samples was lysed by RIPA lysis buffer with protease inhibitors. BCA protein assay kit (ab287853; Abcam, USA) was used to quantify the protein lysates. Before running the gels, equal quantities of the lysates were heated up to 100 °C for 10 min. Western blotting analyses were performed according to the standard procedures. The detailed information of primary antibodies are provided in Additional file 1: Methods.

### Chromatin immunoprecipitation (ChIP)

ChIP assay was handled by a commercial kit (9005; Cell Signaling Technology, USA). The cell homogenate was lysed and incubated with the antibodies against TCF4 (2565; Cell Signaling Technology, USA), histone H3 (H3), and normal rabbit non-immune IgG, at 4 °C overnight, and then incubated with protein A-agarose (1 h). Purified DNA was used for PCR. Human Drp1 primers were as: forward 5′-CCTGCTTCTGCTTCCTAAA-3′ and reverse 5′-CACGCTGATACGAGAATGT-3′.

### Histology and immunohistochemical staining

Parietal peritoneum tissues, embedded in paraffin, were prepared to Sects. (4 μm thickness). Hematoxylin-eosin (H&E) staining was conducted by a standard protocol. For assessing fibrosis extent, at least 10 randomly selected fields of peritoneum tissues from 1 mouse were measured. The thickness of the submesothelial zone was measured, and the average score was calculated. Sirius red staining was performed using the picro-red staining kit (PH1098; Scientific Phygene, China), at room temperature for 2 h.

Paraffin Sects. (4 μm thickness) were performed by immunohistochemical staining with antibodies against fibronectin (FN; F3648; Sigma, USA; 1:200), p16^INK4A^ (sc-1661; Santa Cruz Biotechnology, USA; 1:100), Drp1 (ab184247; Abcam, USA; 1:50), and S100A4/FSP1 (ab218512; Abcam, USA; 1:50).

### Immunofluorescence staining

For immunofluorescence staining, frozen Sects. (4 μm thickness) or coverslides were fixed with 4% paraformaldehyde or cold methanol: acetone (1:1) for 15 min, at room temperature. Furthermore, 0.5% Triton X-100 (10 min) was used for permeabilization and 10% normal donkey serum (1 h) for blocking the non-specific binding. The sections were then incubated with primary antibodies (Additional file 1: Methods) overnight at 4 °C, and then stained by secondary antibodies with Cy2-, Cy3-, or Cy5-conjugated (Jackson Immuno Research Laboratories, USA), and counterstained the nuclei with 4′,6-diamidino-2-phenylindole (DAPI). Sections were viewed by a confocal microscope (Leica TCS SP2 AOBS, Leica Microsystems, Buffalo Grove, IL, USA).

### Statistical analysis

The data are quantified as the mean ± SEM. SPSS 20.0 (SPSS Inc.) software was used to perform statistical analysis. Two-tailed, unpaired Student’s *t*-test was used to calculate the difference between 2 groups. The comparison in multiple groups was calculated by one-way analysis of variance (ANOVA), with Least Significant Difference or Dunnett’s T3 test. *P*-value less than 0.05 is regarded as statistically significant. Spearman correlation analysis was used to assess the correlation among different factors.

## Results

### Peritoneal mesothelial cells undergo senescence fate transition upon long-term PD

**Fig. 1:**
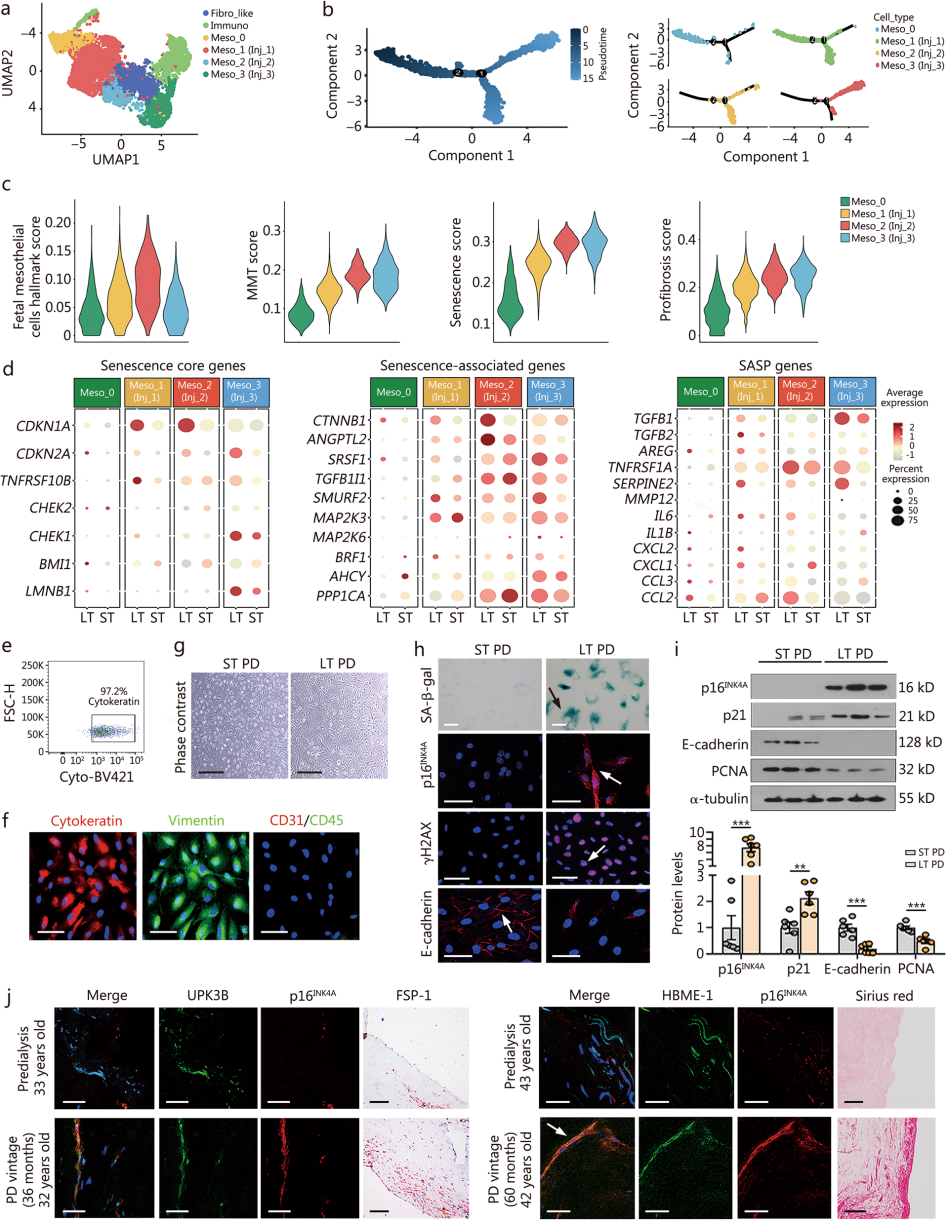
Peritoneal mesothelial cells undergo senescence fate transition upon long-term PD.** a** UMAP visualization showing unsupervised re-clustering of mesothelial cells. **b** Monocle trajectory of mesothelial cells traces a path of pseudotime, and the label with the cell type identity. **c** Violin plots showing scores of fetal mesothelial cells hallmark, MMT, senescence, and profibrosis in the 4 mesothelial subpopulations through the AUCell software. **d** The bubble plots show gene expression of senescence genes in the subclusters of mesothelial cells. **e** Human primary mesothelial cells were sorted through cytokeratin labeling by flow cytometry analysis. **f** Immunofluorescence staining for cytokeratin, vimentin, CD31, and CD45 in primary human mesothelial cells. The cells were co-stained with antibodies against mesothelial cell markers cytokeratin (red) & vimentin (green). The other cells were also stained with antibodies against endothelial cell marker CD31 (red) and leukocyte marker CD45 (green).  Scale bar = 75 μm. **g** Primary human peritoneal mesothelial cells (phase contrast) are shown. Scale bar = 400 μm. **h** Representative staining images show senescence markers SA-β-gal, p16^INK4A^, and γH2AX, and MMT marker E-cadherin in mesothelial cells derived from PD patients for short- or long-term. Arrows indicate positive staining. For SA-β-gal, p16^INK4A^, and E-cadherin staining, scale bar = 50 μm; for γH2AX staining, scale bar = 75 μm. **i** Western blotting showing senescence marker proteins p16^INK4A^, p21, E-cadherin, and PCNA in 2 groups. Quantification analysis is also shown. ^**^P < 0.01, ^***^P < 0.001, by unpaired, two-tailed Student’s *t*-test. *n* = 6. **j** Three-color immunofluorescence staining for DAPI, mesothelial cell marker UPK3B or mesothelial marker HBME-1 and p16^INK4A^ in human peritoneums from predialysis patients and long-term PD patients. Scale bar = 25 μm. FSP-1 staining (scale bar = 100 μm) or Sirius red staining (scale bar = 200 μm) was also performed in the peritoneum from the same PD patients. Fibro-like fibroblast-like cells, Immuno immunocompetent cells, Meso mesothelial cells, MMT mesothelial-mesenchymal transition, SASP senescence-associated secretory phenotype, ST short-term, LT long-term, γH2AX phosphorylated histone H2AX, PCNA proliferating cell nuclear antigen, UPK3B uroplakin 3B, FSP-1 fibroblast-specific protein 1, HBME-1 hector battifora mesothelial cell-1, SA-β-gal senescence-associated β-galactosidase, UMAP Uniform Manifold Approximation and Projection, PD peritoneal dialysis

To explore the profiles of human peritoneal mesothelial cells upon long-term PD, we extracted the publicly available single-cell RNA sequencing (scRNA-seq) dataset (GSE130888) [19] and reanalyzed it. We identified 10 cell clusters on the basis of cell type-specific markers (Additional file 1: Fig. S1a, b). We focused on the mesothelial cell cluster with high expression of mesothelial cell markers such as wilms tumor 1 (*WT1*), mesothelin (*MSLN*), uroplakin 3B (*UPK3B*), and cytokeratin 19 (*KRT19*) (Additional file 1: Fig. S1c). We further extracted the mesothelial cell cluster and subclustered them into 7 subclusters (Additional file 1: Fig. S1d). Based on annotated marker genes and their special genes profiles, we identified 6 distinct cell subtypes (Fig. 1a; Additional file 1: Fig. S1e), i.e., fibroblast-like cells, immuno cells and 4 mesothelial subpopulations (Meso_0–3). We then excluded the mixed fibroblast-like cells and immuno cells, and then performed pseudotemporal ordering and the cell state scores analysis in the 4 mesothelial subpopulations (Meso_0–3). Pseudotemporal ordering analysis showed Meso_0 stepped forward to Meso_1–3 states (Fig. 1b). All Meso_0–3 subgroups showed high expression of fetal mesothelial cells hallmarks, while Meso_1–3 exhibited elevated scores for MMT, cellular senescence, and profibrosis signatures. Therefore, Meso_1–3 subgroups were classified as injury-associated states (Inj_1–3). We observed the highest scores of the above indexes in the Meso_3 (Inj_3) subgroup (Fig. 1c; Additional file 1: Table S4). Furthermore, pseudotime trajectory analysis revealed that extracellular matrix (ECM) and MMT markers were progressively upregulated, along with senescence features in the Meso_3 (Inj_3) cluster (Additional file 1: Fig. S1f). Of interest, we found a rapidly increased feature of MMT at the early stage in Meso_3 (Inj_3) subgroup; however, at the final stage, the MMT feature was slowed down, while the cell senescence feature was upregulated at an extremely rapid speed. At the same time, the ECM production feature is continuously rising (Additional file 1: Fig. S1f). These results suggest the senescence phase is the final endpoint of the MMT phase. We also compared the different ECM profiles of Meso_1–3 subgroups, and found that under long-term PD, Meso_1–3 (Inj_1–3) subgroups produced more ECM components, especially collagens. Of note, the Meso_3 (Inj_3) subgroup had the strongest capability of ECM production (Additional file 1: Fig. S1g).

We then deeply evaluated senescence profiles, including senescence core genes, senescence-associated genes, and SASP genes in the subgroups of mesothelial cells from short- or long-term PD groups. Compared to Meso_0, Meso_1–3 (Inj_1–3) subgroups presented highly enriched profiles of cell senescence (Fig. 1d). Importantly, Meso_3 (Inj_3) subgroup from long-term PD had the strongest senescence signature among the 3 injured mesothelial cell states, which is consistent with pseudotime trajectory analysis (Additional file 1: Fig. S1f). These results highly suggest that mesothelial cells undergo senescence fate transition upon long-term PD.

To further validate these findings, we isolated and cultured primary human mesothelial cells derived from the PD effluent. Patient information is shown in Additional file 1: Table S2. The percentage of mesothelial cells (cytokeratin^+^) was accounted for over 97% in the cells from PD dialysate effluent (Fig. 1e). Furthermore, immunofluorescence analysis showed abundant expression of cytokeratin and vimentin, mesothelial cells markers, but without CD31 and CD45, the markers of endothelial cell and leukocyte (Fig. 1f). Phase contrast imaging revealed that the cells isolated from short-term PD patients had cobblestone morphology, whereas the cells from long-term PD patients turned into spindle-shaped morphology (Fig. 1g). By senescence-associated β-galactosidase (SA-β-gal) staining, a widely used marker for senescent cells, we found excessive staining for SA-β-gal in long-term PD-induced mesothelial cells (Fig. 1h). Moreover, these cells exhibited more expression of p16^INK4A^ and phosphorylated histone H2 (γH2AX), the other important senescence markers, and loss of E-cadherin, an indicator for MMT process (Fig. 1h). Western blotting analysis also showed that long-term PD upregulated p16^INK4A^ and p21 expression but downregulated the expression of E-cadherin and proliferating cell nuclear antigen (PCNA), a cell proliferation marker (Fig. 1i). In PDF-treated mice, p16^INK4A^ and γH2AX were also highly increased in mesothelial cells (indicated by UPK3B) (Additional file 1: Fig. S2a).

We also measured the expression levels of p16^INK4A^ and γH2AX in peritoneum tissues from predialysis and long-term PD patients at similar ages. Patient information is shown in Additional file 1: Table S1. Mesothelial cells were identified using markers such as WT1, UPK3B, HBME-1, and cytokeratin 18 (Fig. 1j; Additional file 1: Fig. S2c, d). Compared with predialysis patients, long-term PD patients exhibited significantly higher levels of p16^INK4A^ and γH2AX in mesothelial cells (Fig. 1j**; **Additional file 1: Fig. S2b, c) and loss of E-cadherin expression (Additional file 1: Fig. S2d). Histological analysis using fibroblast-specific protein 1 (FSP-1) and Sirius red staining revealed enhanced fibroblast activation and excessive collagen deposition in the submesothelial zone, accompanied by increased peritoneum thickness in long-term PD patients (Fig. 1j).

### β-catenin and transforming growth factor-β (TGF-β) signaling increase in mesothelial cells along with PD duration

**Fig. 2:**
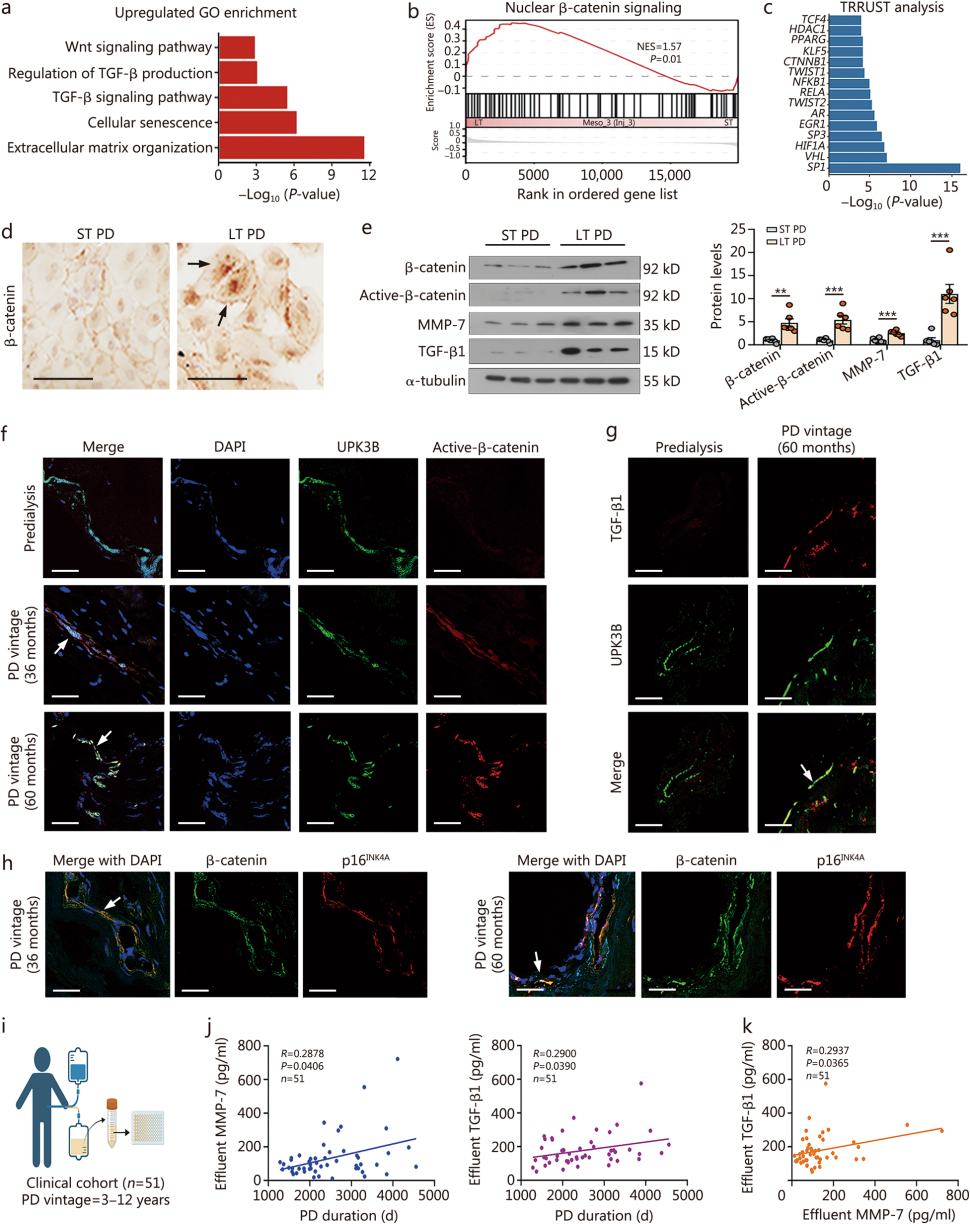
β-catenin and TGF-β signaling increase in mesothelial cells along with PD duration. **a** The upregulated GO enrichment items of long- vs. short-term PD in the injury state 3 subtype mesothelial cells. **b** GSEA analysis shows the gene set of nuclear β-catenin signaling in short- and long-term PD group. **c** The upregulated TRRUST enrichment items of long- vs. short-term PD in the injury state 3 subtype. **d** Human primary mesothelial cells were immunostained for β-catenin in 2 groups. Arrows indicate positive staining. Scale bar = 100 μm. **e** Representative Western blotting showing the expression of β-catenin, active-β-catenin, MMP-7, and TGF-β1. Quantitative data are also shown. ^**^P < 0.01, ^***^P < 0.001, by unpaired, two-tailed Student’s *t*-test (*n* = 6). **f** Three-color immunofluorescence staining for DAPI, UPK3B (mesothelial marker), and active-β-catenin in human peritoneum from a predialysis patient and 2 long-term PD patients. Arrows indicate positive staining. Scale bar = 25 μm. **g** TGF-β1 is co-stained with UPK3B in a predialysis patient and a long-term PD patient. The white arrow indicates positive staining. Scale bar = 25 μm. **h** Three-color immunofluorescence staining for DAPI, β-catenin, and p16^INK4A^ in human peritoneums after long-term treatment of PD. White arrows indicate β-catenin and p16^INK4A^ are colocalized. Scale bar = 25 μm. **i** The graph shows that 51 long-term PD patients’ effluent was collected and then analyzed by ELISA. **j** Correlation between effluent MMP-7 or TGF-β1 and PD duration in long-term PD patients. Spearman correlation coefficient (*R*) value and *P*-value are shown. **k** Correlation between effluent MMP-7 and effluent TGF-β1. The Spearman correlation coefficient (*R*) value and *P*-value are shown. GO Gene Ontology, TRRUST Transcriptional Regulatory Relationships Unraveled by Sentence-based Text-mining, GSEA Gene Set Enrichment Analysis, NES normalized enrichment score, ST short-term, LT long-term, MMP-7 metalloproteinase-7, TGF-β1 transforming growth factor-β1, UPK3B uroplakin 3B, PD peritoneal dialysis, DAPI 4′,6-diamidino-2-phenylindole

To elucidate the potential molecular mechanisms, Gene Ontology (GO), Gene Set Enrichment Analysis (GSEA), and Transcriptional Regulatory Relationships Unraveled by Sentence-based Text-mining (TRRUST) enrichment analyses were performed in the Meso_3 (Inj_3) subgroup, which represents the most senescent state among the identified mesothelial cell subpopulations. As illustrated in Fig. 2a, b, both GO and GSEA analyses revealed Wnt and TGF-β signaling pathways, and β-catenin signaling were significantly activated in the Meso_3 (Inj_3) subgroup derived from long-term PD patients, coinciding with the senescent phenotype and ECM organization. From TRRUST analysis, we observed 15 transcription factors that significantly participated in gene regulation in the Meso_3 (Inj_3) subgroup. Of interest, *CTNNB1* (the gene encoding β-catenin) and its downstream transcription factor *TCF4,* the downstream executors of Wnt/β-catenin signaling*,* were enriched among them (Fig. 2c), suggesting their regulatory roles.

We subsequently performed immunostaining for β-catenin in primary human mesothelial cells. β-catenin was remarkably induced in the nucleus of mesothelial cells isolated from long-term PD patients (Fig. 2d), suggesting the activation of β-catenin. Western blotting analyses further confirmed the increased expression levels of β-catenin, active-β-catenin, matrix metalloproteinase-7 (MMP-7), a β-catenin downstream target, and TGF-β1 in long-term PD-induced mesothelial cells (Fig. 2e).

We further assessed β-catenin expression in the peritoneum of patients undergoing PD. As shown in Fig. 2f and Additional file 1: Fig. S2e, f, active-β-catenin and β-catenin were increased in mesothelial cells along with PD duration. Interestingly, TGF-β1 expression was significantly increased in the mesothelial layer of long-term PD patients, whereas its expression remained minimal in predialysis patients (Fig. 2g; Additional file 1: Fig. S2g). Furthermore, in the mesothelial layer, β-catenin expression largely colocalized with p16^INK4A^. This expression pattern was exacerbated under longer PD duration (Fig. 2h).

We also collected effluent from PD patients (Fig. 2i). Patients’ information is shown in Additional file 1: Table S3. Notably, MMP-7, the β-catenin downstream target, and TGF-β1 in effluent were positively increased following the duration of PD (Fig. 2j). Both MMP-7 and TGF-β1 were also positively correlated with each other (Fig. 2k). These results further demonstrated β-catenin activation is associated with mesothelial cell senescence, concomitantly with TGF-β activation.

### β-catenin-induced mitochondrial fission contributes to mesothelial cell senescence

**Fig. 3:**
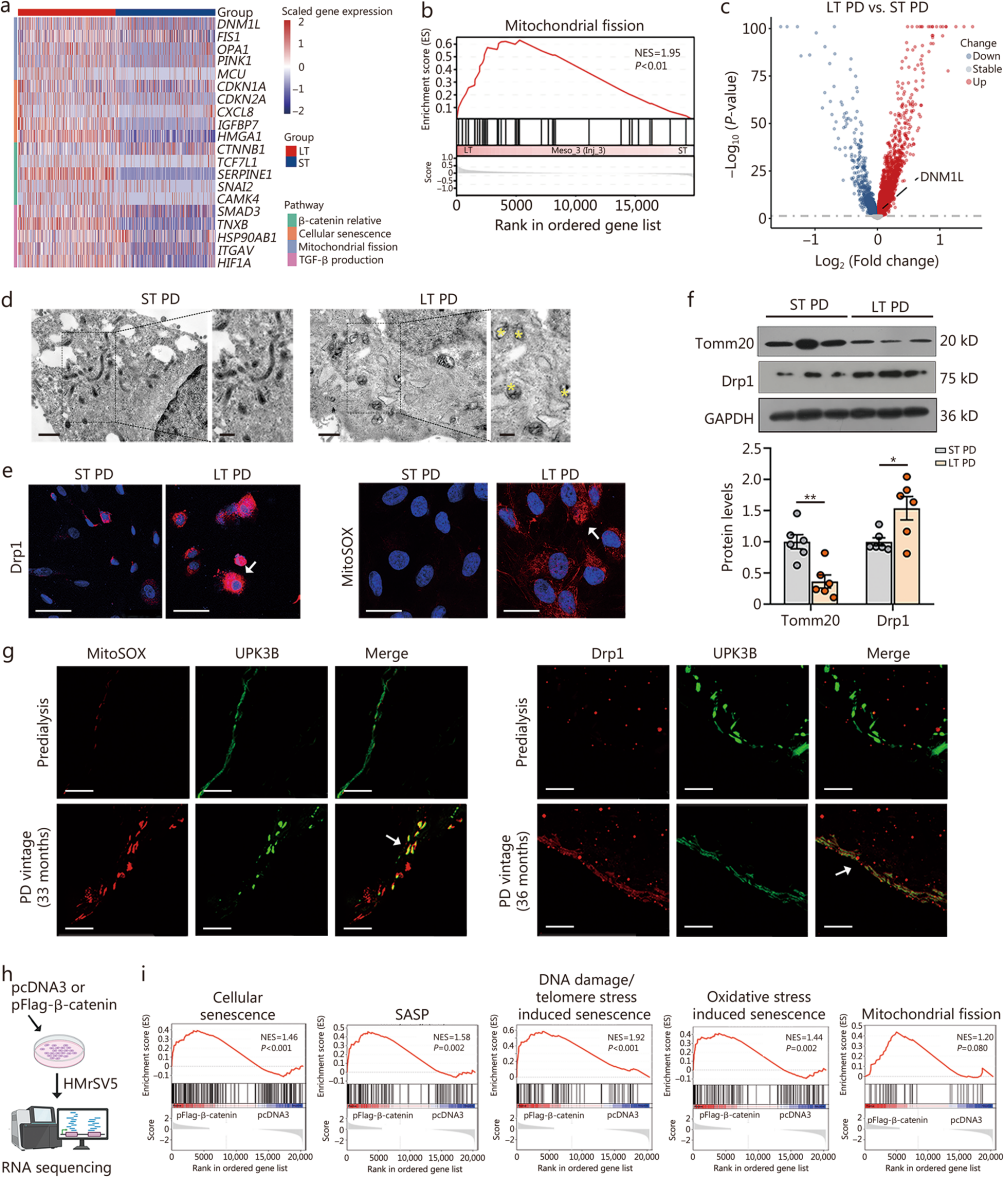
β-catenin-triggered mitochondrial fission contributes to mesothelial cell senescence. **a** The heatmap plots show gene expression of β-catenin relative signaling, cellular senescence, mitochondrial fission, and TGF-β production between 2 groups in injury state 3 subtype. **b** GSEA analysis shows the gene set of mitochondrial fission in short- and long-term PD groups. **c** Volcano plot shows the differentiation of expressed genes of the 2 groups; the symbol-marked dot indicates DNM1L (coding Drp1). **d** Representative TEM micrographs of human primary mesothelial cells show mitochondria in 2 groups. Scale bar = 1.0 μm (left) and 0.5 μm (right). Yellow stars indicate vacuolization and fragmentation of mitochondria. **e** Representative images show the expression of Drp1 and mtROS, as detected by immunofluorescence staining for Drp1 and MitoSOX staining, respectively, in human primary mesothelial cells in 2 groups. Arrows indicate positive staining. Scale bar = 50 μm. **f** Western blotting analyses showing Tomm20 and Drp1 expression. Quantitative data are shown. ^*^P < 0.05, ^**^P < 0.01, by unpaired, two-tailed Student’s *t*-test (*n* = 6). **g** Co-staining of MitoSOX and UPK3B was performed in human peritoneums from a predialysis patient and a long-term PD patient. Co-staining of Drp1 and UPK3B was also performed. White arrows indicate positive staining. Scale bar = 25 μm. **h** Flow chart shows the experimental design and procedures. HMrSV5 cells were transfected with either a β-catenin plasmid or an empty vector, and then subjected to RNA sequencing.** i** GSEA analysis shows that cellular senescence, SASP, DNA damaged/telomere stress induced senescence, oxidative stress induced senescence, and mitochondrial fission were mainly enriched in the pFlag‐β-catenin group compared with the pcDNA3 group.* P*-values were labeled in the plots. NES normalized enrichment score, ST short-term, LT long-term, GSEA Gene Set Enrichment Analysis, Drp1 dynamin-related protein 1, TEM transmission electron microscope, Tomm20 mitochondrial import receptor subunit TOM20 homolog, SASP senescence-associated secretory phenotype, TGF-β transforming growth factor-β, PD peritoneal dialysis, UPK3B uroplakin 3B, mtROS mitochondrial reactive oxygen species, MitoSOX mitochondrial superoxide indicator

To elucidate the pivotal mechanisms of mesothelial cell senescence, we conducted a heatmap analysis in the Meso_3 (Inj_3) subgroup. As shown, in long-term PD group, β-catenin, cell senescence signaling, and TGF-β production were increased in mesothelial cells, accompanied by the upregulation of genes associated with mitochondrial fission (Fig. 3a). GSEA and volcano plot analysis further confirmed that the mitochondrial fission pathway, as well as *DNM1L* (which encodes Drp1, the key regulator of mitochondrial fission), were markedly enriched and upregulated (Fig. 3b, c).

From transmission electron microscope (TEM) analysis, we observed fragmented mitochondria highly increased in long-term PD-affected mesothelial cells (Fig. 3d). Staining showed Drp1 and mitochondrial reactive oxygen species (mtROS) were increased in mesothelial cells under long-term PD conditions (Fig. 3e). Western blotting analyses further showed decreased mitochondrial import receptor subunit TOM20 homolog (Tomm20), mitochondrial outer membrane marker, and upregulated Drp1 in long-term group. (Fig. 3f). Costaining revealed increased mtROS production and Drp1 expression in the mesothelial cell layer in long-term PD patients (Fig. 3g).

To test the involvement of β-catenin in mesothelial cell injury, the human peritoneal mesothelial cell line (HMrSV5 cells) was overexpressed with a β-catenin-expressing plasmid (pFlag-β-catenin). RNA sequencing showed ectopic β-catenin upregulated a variety of senescence-related pathways, accompanied by mitochondrial fission (Fig. 3h, i; Additional file 1: Fig. S3a). Western blotting and heatmap analysis showed β-catenin triggered p16^INK4A^ and γH2AX upregulation, and other cell senescence and mitochondrial fission-related genes (Additional file 1: Fig. S3b, c). In summary, our results indicated that β-catenin induces mesothelial cell senescence and mitochondrial fission, which is involved in the process of cell senescence.

### β-catenin triggers mesothelial cell senescence by promoting Drp1

**Fig. 4:**
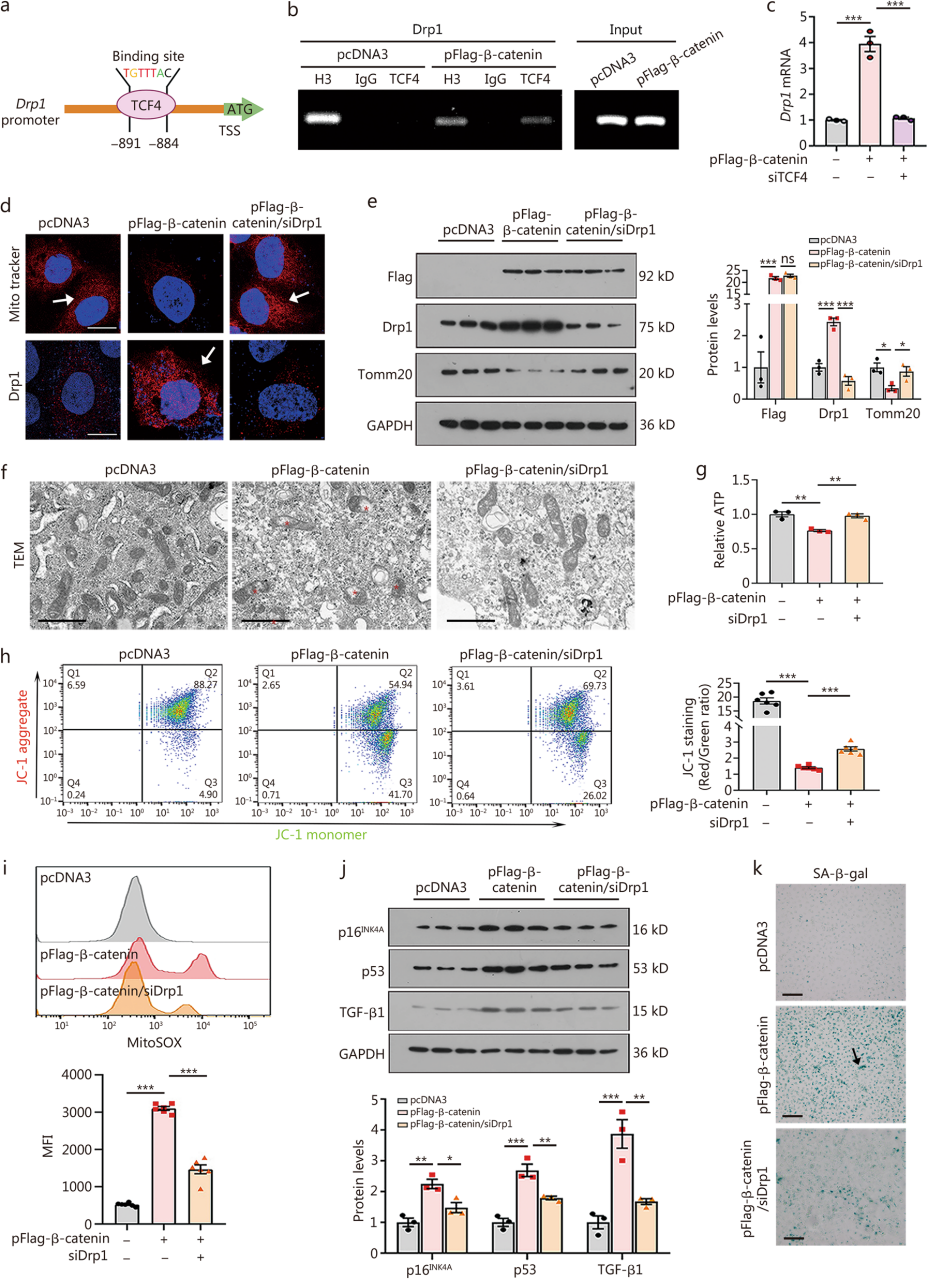
β-catenin triggers mesothelial cell senescence by promoting Drp1. **a** The binding site of TCF4 in *Drp1* promoter is shown. **b** ChIP assay showed TCF4 binds to the gene promoter region of *Drp1*. β-catenin triggered the binding of TCF4 to the *Drp1* promoter after ectopic expression for 48 h. Antibodies against H3, non-immune IgG, and TCF4 were used for immunoprecipitation. Input control was operated on total genomic DNA. **c** mRNA expression of *Drp1* in different groups. ^***^P < 0.001, by one-way ANOVA followed by the Least Significant Difference test (*n* = 3). **d** Mito tracker and Drp1 staining in different groups. Arrows indicate positive staining. Scale bar = 10 μm. **e** Western blotting and quantitative data of Flag, Drp1, and Tomm20. ^*^P < 0.05, ^***^P < 0.001, by one-way ANOVA followed by the Least Significant Difference test (*n* = 3). **f** Representative TEM micrographs of mitochondria in different groups. Red stars indicate vacuolization and fragmentation in mitochondria. Scale bar = 1 μm. **g** ATP contents in different groups are shown. ^**^P < 0.01, by one-way ANOVA followed by the Least Significant Difference test (*n* = 3). **h** JC-1 staining showed β-catenin decreases MMP, but interference of Drp1 inhibits it. Graphical representation of the average red/green fluorescence ratio. ^***^P < 0.001, by one-way ANOVA followed by Dunnett’s T3 procedure test (*n* = 6). **i** Flow cytometry analysis of MitoSOX-probe-stained HMrSV5 cells in different groups. ^***^P < 0.001, by one-way ANOVA followed by the Least Significant Difference test (*n* = 6). **j** Western blotting showing p16^INK4A^, p53 and TGF-β1. Quantitative data are shown. ^*^P < 0.05, ^**^P < 0.01, ^***^P < 0.001, by one-way ANOVA followed by the Least Significant Difference test (*n* = 3). **k** The SA-β-gal staining in different groups. Arrow indicates positive staining. Scale bar = 200 μm. ns not significance, Drp1 dynamin-related protein 1, TCF4 transcription factor 4, H3 histone H3, Tomm20 mitochondrial import receptor subunit TOM20 homolog, siDrp1 dynamin-related protein 1 small interfering RNA, TEM transmission electron microscope, ATP adenosine triphosphate, MMP mitochondrial membrane potential, MFI median fluorescence intensity, TGF-β1 transforming growth factor-β1, SA-β-gal senescence-associated β-galactosidase, TSS transcriptional start site, ATG adenine–thymine-guanine

By bioinformatics analysis, a perfect binding site of TCF4, a key transcription factor for β-catenin transduction [35], was shown in the promoter region of *Drp1* (Fig. 4a). ChIP-qPCR analysis showed β-catenin induced the binding of TCF4 with *Drp1* promoter (Fig. 4b). Quantitative PCR further demonstrated β-catenin induced the upregulation of *Drp1*, while this effect was reversed by interference of TCF4 (Fig. 4c).

Mito tracker and Drp1 staining revealed that ectopic β-catenin diminished mitochondrial mass and triggered Drp1 upregulation; however, these effects were suppressed by Drp1 interference (Fig. 4d**)**. Similar results were observed when Drp1 was examined by Western blotting analyses (Fig. 4e). Furthermore, knockdown of *Drp1* preserved the expression of Tomm20 and restored mitochondrial morphology (Fig. 4e, f). ATP assay, JC-1 staining, and MitoSOX staining revealed that knockdown of *Drp1* preserved ATP production and mitochondrial membrane potential (MMP), and blocked mtROS in β-catenin-overexpressed cells (Fig. 4g-i). Furthermore, inhibition of Drp1 significantly suppressed the upregulation of p16^INK4A^, p53, and TGF-β1, which was induced by β-catenin overexpression, and reduced SA-β-gal activity (Fig. 4j, k). These results suggest that Drp1 strongly contributes to β-catenin-induced mesothelial cell senescence.

### β-catenin induces SASP secretion to mediate mesothelial cells communicating with fibroblasts

**Fig. 5:**
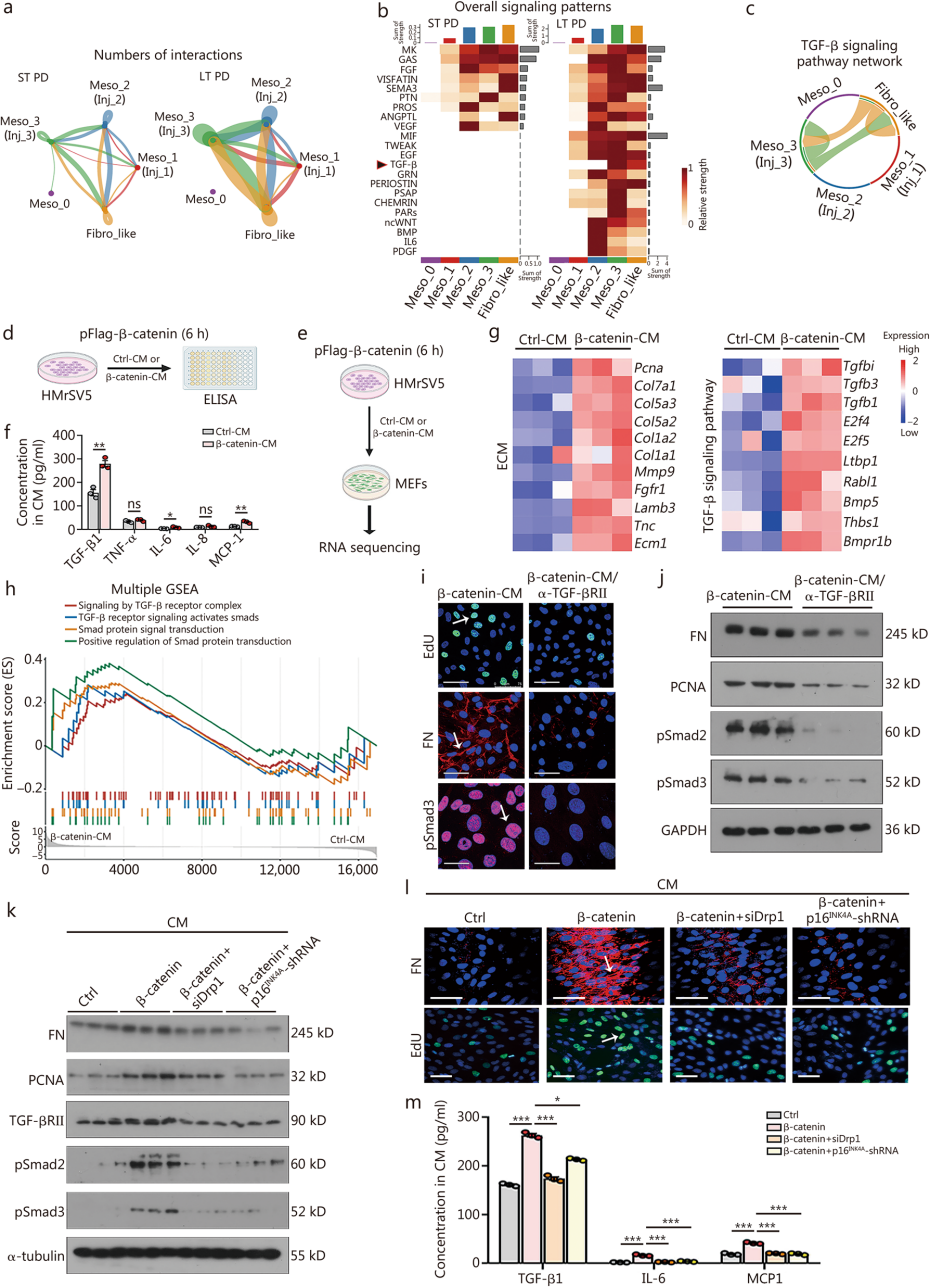
β-catenin induces SASP secretion to mediate mesothelial cells communicating with fibroblasts. **a** The chordal graph of cell communication of mesothelial cell, injury state subtypes, and fibroblast-like cell in short- or long-term PD groups. **b** The heatmap plots show the strength of overall enriched signals (outgoing and incoming) within each cluster. **c** The chord graph shows the intercellular communication network for TGF-β signaling. **d** The experimental design is shown. β-catenin or empty vector was overexpressed for 6 h. The supernatant was collected (β-catenin-CM or Ctrl-CM) and assessed by ELISA. **e** Experimental design. HMrSV5 cells were transfected with either a β-catenin plasmid or an empty vector for 6 h, after which the serum-free supernatant was collected and used as β-catenin-CM or Ctrl-CM. Subsequently, MEFs were treated with β-catenin-CM or Ctrl-CM and subjected to RNA sequencing. **f** Expression of SASP factors in CM from different groups. ^*^P < 0.05, ^**^P < 0.01, by unpaired, two-tailed Student’s* t*-test (*n* = 3). **g** Heatmap showing the core gene expression of ECM and TGF-β signaling pathway in Ctrl-CM-treated MEFs compared with β-catenin-CM-treated MEFs. The color gradient refers to the scale expression level, standardizing gene expression using the scale function. The color gradient from dark blue to dark red refers to the gene expression level from low to high (*n* = 3). **h** Multi GSEA enrichment analysis shows that signaling by TGF-β receptor complex, TGF-β receptor signaling activates Smad, Smad signal transduction, and positive regulation of Smad were mainly enriched in β-catenin-CM-treated MEF cells. **i** EdU staining, FN staining, and pSmad3 staining in 2 groups. Arrows indicate positive staining. For EdU staining, scale bar = 75 μm; for FN staining, scale bar = 50 μm; for pSmad3 staining, scale bar = 25 μm. **j** Western blotting analyses show FN, PCNA, pSmad2, and pSmad3 expression levels in 2 groups. **k** Western blotting of FN, PCNA, TGF-βRII, pSmad2, and pSmad3. **l** FN and EdU staining in different groups. Arrows indicate positive staining. For FN staining, scale bar = 50 μm; for EdU staining, scale bar = 100 μm. **m** Expression of SASP factors in CM from different groups. ^*^P < 0.05, ^***^P < 0.001, by one-way ANOVA followed by the Least Significant Difference test (*n* = 3). ns not significance, ST short-term, LT long-term, PD peritoneal dialysis, Fibro_like fibroblast-like cells, Meso mesothelial cells, PCNA proliferating cell nuclear antigen, pSmad2 phosphorylated Smad 2, pSmad3 phosphorylated Smad 3, TGF-βRII transforming growth factor-β receptor type II, siDrp1 dynamin-related protein 1 small interfering RNA, p16^INK4A^-shRNA p16^INK4A^ short hairpin RNA, TNF-α tumor necrosis factor α, IL-6 interleukin-6, IL-8 interleukin-8, MCP-1 monocyte chemoattractant protein-1, FN fibronectin, SASP senescence-associated secretory phenotype, CM conditioned medium, TGF-β transforming growth factor-β, ELISA enzyme-linked immunosorbent assay, GSEA Gene Set Enrichment Analysis, MEFs mouse embryonic fibroblasts, ECM extracellular matrix, EdU 5-Ethynyl-2'-deoxyuridine

We hypothesized that β-catenin activates TGF-β1 to facilitate intercellular communication. To test this hypothesis, we analyzed cell–cell interaction (CCI) activity between mesothelial cells and fibroblast-like cells. As shown in Fig. 5a, in the long-term PD group, injured mesothelial cells [Meso_1 (Inj_1) – Meso_3 (Inj_3)] exhibited increased interactions with fibroblast-like cells (Fibro_like), particularly in the Meso_3 (Inj_3) subgroup, whereas naive mesothelial cells (Meso_0) showed no interactions with fibroblast-like cells. We further analyzed the sender-receiver relationships of signaling molecules and found that TGF-β signaling was significantly enriched in signals received by fibroblast-like cells in the long-term group (Fig. 5b). The TGF-β signaling pathway network also revealed strong connectivity between Inj_3 mesothelial cells and fibroblast-like cells (Fig. 5c).

Conditioned medium (CM), the supernatant of β-catenin-overexpressed mesothelial cells, was used to treat MEFs (Fig. 5d, e). We found that several SASP molecules, including TGF-β1, interleukin (IL)-6, and monocyte chemoattractant protein-1 (MCP-1), were upregulated in β-catenin CM, whereas tumor necrosis factor-α (TNF-α) or IL-8 levels remained unchanged (Fig. 5f). Notably, among these SASP factors, TGF-β1 was the most significantly induced in β-catenin-CM.

Furthermore, RNA sequencing revealed that β-catenin-CM-treated MEFs exhibited significant upregulation of ECM-related genes and activation of the TGF-β signaling pathway (Fig. 5g). GSEA further confirmed that β-catenin-CM activated the TGF-β signaling cascade and its downstream pathway of Smad (Fig. 5h). We also observed that β-catenin-CM-induced MEF cell proliferation, FN expression, as well as phosphorylation of Smad3 and Smad2 were markedly suppressed by a neutralizing antibody targeting the TGF-β receptor II (TGF-βRII) (Fig. 5i, j; Additional file 1: Fig. S4a). To further validate this mechanism, we treated MEF cells with TGF-β1 at a concentration of 250 pg/ml, which mimics the TGF-β1 levels found in β-catenin-CM. Notably, even this low concentration of TGF-β1 was sufficient to promote cell proliferation (Additional file 1: Fig. S4b). These results indicate that TGF-β1 is the primary mediator of β-catenin-CM-induced communication between mesothelial cells and fibroblasts.

Moreover, knockdown of *Drp1* or *p16*^*INK4A*^ significantly suppressed TGF-β/Smad signaling, cell proliferation, and FN expression in β-catenin-CM-treated MEFs (Fig. 5k, l; Additional file 1: Figs. S4c-e). Additionally, ELISA analyses demonstrated that the elevated levels of TGF-β1, IL-6, and MCP-1 in β-catenin-CM were markedly reduced following *Drp1* or *p16*^*INK4A*^ silencing (Fig. 5m).

### Knockout of β-catenin in mesothelial cells ameliorates mesothelial cell senescence in PDF mice

**Fig. 6:**
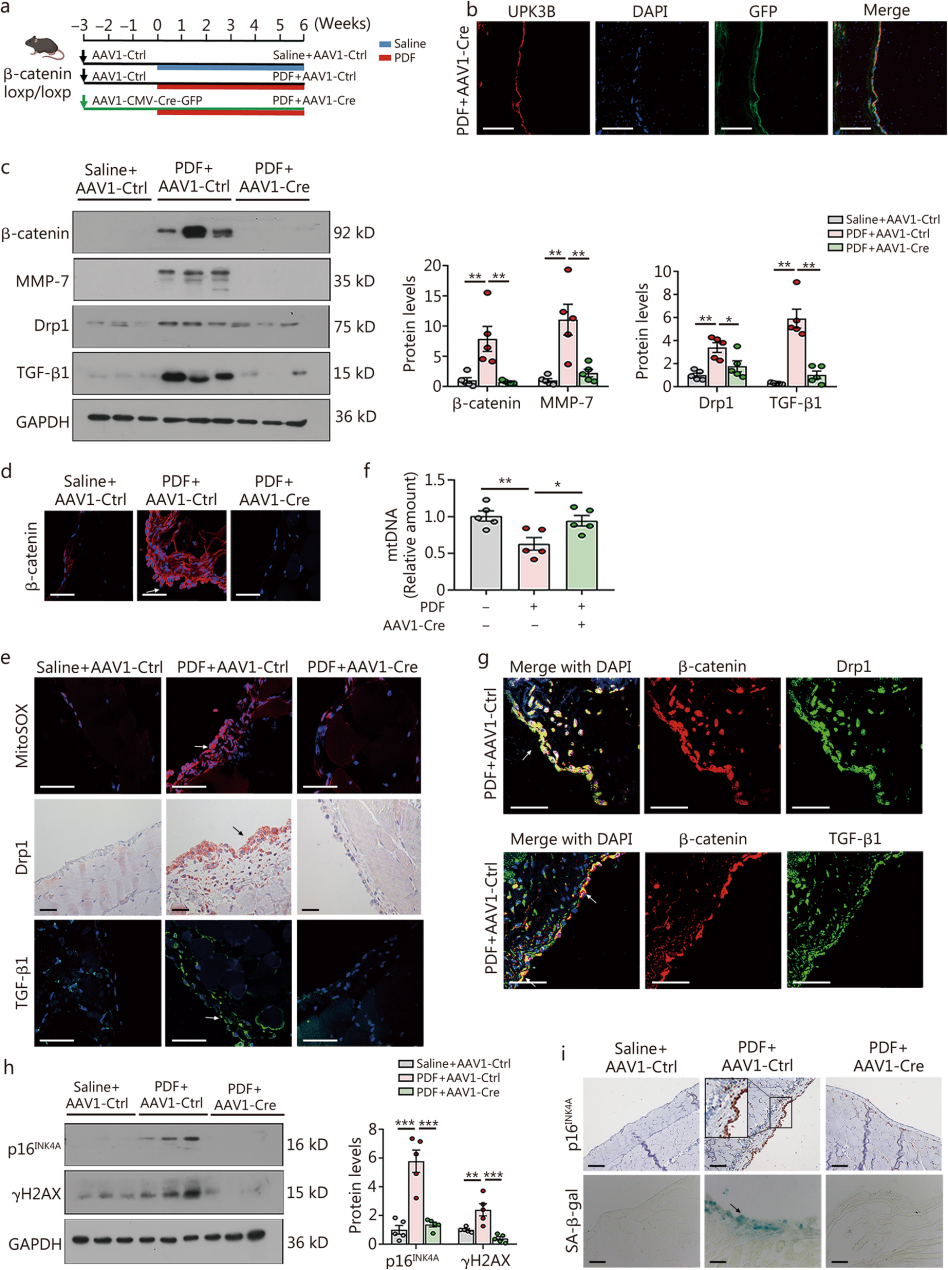
Knockout of β-catenin in mesothelial cells ameliorates mesothelial cell senescence in PDF mice. **a** Experimental design. β-catenin^loxP/loxP^ mice were injected with AAV1-CMV-Cre-GFP or AAV1-control viruses 3 weeks prior to the initiation of the 6-week PDF treatment. **b** Colocalization of UPK3B and GFP in the peritoneum of a mouse treated with AAV1-CMV-Cre. Scale bar = 50 μm. **c** Western blotting of β-catenin, MMP-7, Drp1 and TGF-β1. Quantitative data are shown. ^*^P < 0.05, ^**^P < 0.01, by one-way ANOVA followed by the Least Significant Difference test (*n* = 5). **d** β-catenin staining in 3 groups. Arrow indicates positive staining. Scale bar = 50 μm. **e** MitoSOX, Drp1, and TGF-β1 staining in different groups. Arrows indicate positive staining. Scale bar = 50 μm. **f** Mitochondrial DNA (mtDNA) levels were analyzed. Mitochondrial NADH dehydrogenase subunit1 (ND1) was assessed to represent mtDNA. Ribosomal protein s18 (RSP18) was used for normalization. ^*^P < 0.05, ^**^P < 0.01, by one-way ANOVA followed by the Least Significant Difference test (*n* = 5). **g** Colocalization of β-catenin (red) with Drp1 (green) or TGF-β1 (green) in PDF + AAV1-Ctrl treated mice. White arrows indicate positive staining. Scale bar = 50 μm. **h** Western blotting analyses of p16^INK4A^ and γH2AX. Quantitative data are shown. ^**^P < 0.01, ^***^P < 0.001, by one-way ANOVA followed by the Least Significant Difference test (*n* = 5).** i** Staining of p16^INK4A^ and SA-β-gal activity. Arrow or box indicates positive staining. For p16^INK4A^ staining, scale bar = 100 μm; for SA-β-gal staining, scale bar = 50 μm. PDF peritoneal dialysis fluid, UPK3B uroplakin 3B, GFP green fluorescent protein, MMP-7 metalloproteinase-7, Drp1 dynamin-related protein 1, TGF-β1 transforming growth factor-β1, γH2AX phosphorylated histone H2, SA-β-gal senescence-associated β-galactosidase, AAV1-CMV-Cre adeno-associated virus serotype 1-carried the Cre recombinase under CMV promoter, DAPI 4′,6-diamidino-2-phenylindole, MitoSOX mitochondrial superoxide indicator

To further investigate the role of β-catenin in peritoneal fibrosis, β-catenin knockout mice were generated by a single intraperitoneal injection of adeno-associated virus expressing Cre-recombinase with GFP labeling (AAV1-Cre-GFP) into β-catenin floxed mice. These mice were subsequently treated with 4.25% PDF for 6 weeks (Fig. 6a). As shown in Fig. 6b, AAV1-Cre-GFP injection led to a high infection efficiency in the peritoneal mesothelial layer, as indicated by the colocalization of UPK3B and GFP in the peritoneum. Efficient deletion of β-catenin was further confirmed by Western blotting analyses and immunofluorescence staining of peritoneal tissues (Fig. 6c, d). PDF treatment induced mtROS production, accompanied by increased expression of Drp1 and TGF-β1. However, these effects were significantly attenuated in β-catenin knockout mice (Fig. 6c, e). Moreover, mtDNA levels were elevated in β-catenin knockout mice, compared to those treated with PDF + AAV1-Ctrl (Fig. 6f). Interestingly, Drp1 and TGF-β1 strongly colocalized with β-catenin in the mesothelial layer of mice treated with PDF + AAV1-Ctrl (Fig. 6g), further supporting the critical role of the β-catenin-Drp1-TGF-β1 axis in peritoneal injury.

We further evaluated the expression of p16^INK4A^ and γH2AX, two well-established markers of cellular senescence, and found that their levels were markedly reduced in β-catenin knockout mice (Fig. 6h). Consistently, both p16^INK4A^- and SA-β-gal-positive cells were significantly increased in PDF-treated mice but were notably decreased in β-catenin knockout mice (Fig. 6i). Collectively, these results further demonstrate that β-catenin plays a pivotal role in mesothelial cell senescence and is closely associated with mitochondrial fission and TGF-β1 secretion.

### Knockout of β-catenin disrupts the cell communications between mesothelial cells and fibroblasts, and alleviates peritoneal fibrosis

To further demonstrate that β-catenin mediates the interaction of mesothelial cells and fibroblasts, multicolor immunofluorescence staining was conducted using antibodies against UPK3B (a mesothelial cell marker), E-cadherin (an MMT indicator), and FSP-1 (a fibroblast marker). As shown in Additional file 1: Fig. S5a, PDF-treated mice exhibited the impaired linear distribution of UPK3B and loss of E-cadherin expression in the mesothelial cell layer, along with a marked increase in FSP-1 levels, which were shown in the submesothelial zone. Whereas these changes were reversed in *β-catenin* knockout mice. Moreover, immunostaining revealed that TGF-β1 expression was upregulated in the mesothelial layer of PDF-treated mice, accompanied by a significant accumulation of α-smooth muscle actin (α-SMA) + myofibroblasts beneath the mesothelial layer. In contrast, these effects were largely reduced in β-catenin knockout mice upon PDF treatment (Additional file 1: Fig. S5b).

We further performed costaining of FSP-1 with TGF-βRII or p-Smad3, which are key components of the TGF-β signaling pathway [36]. These markers were highly co-expressed in PDF-treated mice, indicating activation of the TGF-β/Smad signaling in fibroblasts. However, this co-expression was significantly reduced in β-catenin knockout mice (Additional file 1: Fig. S5c, d).

Histological examination and immunostaining further revealed that β-catenin deficiency attenuated peritoneal thickness and fibroblast activation in PDF mice (Additional file 1: Fig. S5e). Consistently, the expression levels of fibrotic proteins, including FN and α-SMA, were markedly suppressed in β-catenin knockout mice (Additional file 1: Fig. S5f).

We also treated PDF mice with KYA1797K (10 mg/kg), a small molecule promoting β-catenin degradation [37]. Notably, KYA1797K decreased β-catenin expression, significantly inhibited Drp1, p16^INK4A^, and TGF-β expression. Furthermore, KYA1797K treatment effectively retarded fibroblast activation and peritoneal fibrosis (Additional file 1: Fig. S6).

Collectively, these findings suggest that β-catenin plays a central role in peritoneal fibrosis by mediating the crosstalk between mesothelial cells and fibroblasts.

### Senolytic therapy ameliorates peritoneal fibrosis

**Fig. 7:**
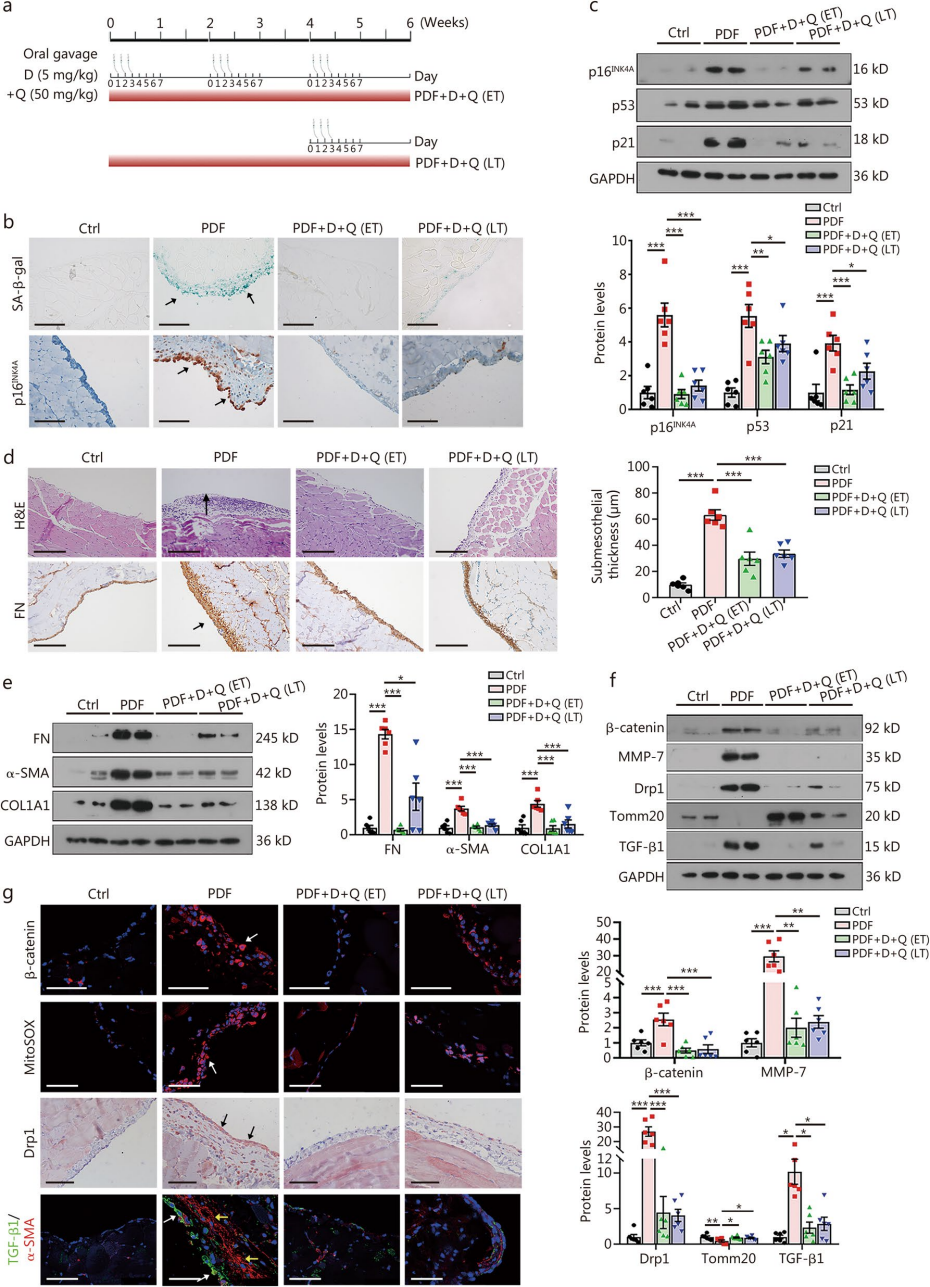
Senolytic therapy ameliorates peritoneal fibrosis. **a** Experimental design. Gavage needles indicate that D + Q were administered by oral gavage to PDF mice. Mice were given D + Q on days 1–3, every 2 weeks, 1 cycle every 2 weeks for 6 weeks. The other mice were given 3 times at the beginning of the 5th week of PDF injection. **b** SA-β-gal staining in parietal peritoneum. Blue-green staining is considered positive. Scale bar = 200 μm. Paraffin sections of parietal peritoneum were stained for p16^INK4A^. p16^INK4A^-positive cells were mainly localized in the mesothelial layer in mice treated with PDF, but were suppressed by D + Q. Scale bar = 100 μm. Arrows indicate positive staining. **c** Western blotting of p16^INK4A^, p53, and p21. Quantitative data are shown. ^*^P < 0.05, ^**^P < 0.01, ^***^P < 0.001, by one-way ANOVA followed by the Least Significant Difference test (*n* = 6). **d** H&E staining and FN staining of peritoneum tissue in 4 groups are indicated. Arrows indicate positive staining. Scale bar = 200 μm. Peritoneal thickness is shown. ^***^P < 0.001, by one-way ANOVA followed by the Least Significant Difference test (*n* = 6). **e** Western blotting and quantitative data of FN, COL1A1 and a-SMA. ^*^P < 0.05, ^***^P < 0.001, for FN, by one-way ANOVA followed by Dunnett’s T3 procedure test; for COL1A1 and α-SMA, by one-way ANOVA followed by the Least Significant Difference test (*n* = 6). **f** Western blotting analyses and quantitative data of β-catenin, MMP-7, Drp1, Tomm20 and TGF-β1. ^*^P < 0.05, ^**^P < 0.01, ^***^P < 0.001, for β-catenin and Tomm20, by one-way ANOVA followed by the Least Significant Difference test; for MMP-7, Drp1 and TGF-β1, by one-way ANOVA followed by Dunnett’s T3 procedure test (*n* = 6)** g** Peritoneal expression of β-catenin, mtROS production, Drp1, and TGF-β1/α-SMA in 4 groups. Arrows indicate positive staining. For β-catenin, MitoSOX, and TGF-β1/α-SMA staining, scale bar = 50 μm; for Drp1 staining, scale bar = 100 μm. D + Q dasatinib plus quercetin, ET early treatment, LT late treatment, FN fibronectin, COLlA1 collagen type alpha 1 chain, α-SMA α-smooth muscle actin, H&E hematoxylin–eosin staining, MMP-7 metalloproteinase-7, Drp1 dynamin-related protein 1, TGF-β1 transforming growth factor-β1, SA-β-gal senescence-associated β-galactosidase, PDF peritoneal dialysis fluid, Tomm20 mitochondrial import receptor subunit TOM20 homolog, MitoSOX mitochondrial superoxide indicator

We treated PDF mice with dasatinib and quercetin (D + Q), which are established senolytic agents known to eliminate senescent cells selectively [38]. D + Q were administered orally at the indicated time (Fig. 7a). As shown in Fig. 7b, c, PDF-treated mice exhibited a pronounced senescence phenotype, characterized by elevated SA-β-gal activity and increased expression of p16^INK4A^, p53, and p21. Both early and late D + Q treatments significantly reduced markers of cellular senescence. Furthermore, histological analysis revealed that D + Q treatment attenuated the PDF-induced increase in peritoneal thickness (Fig. 7d). Western blotting analysis confirmed that the expression of fibrotic markers, including FN, α-SMA, and collagen type alpha 1 chain (COL1A1), was significantly suppressed in both D + Q-treated groups (Fig. 7e).

We also evaluated the effect of D + Q on the β-catenin-Drp1-TGF-β1 signaling axis. Notably, both early and late therapies effectively inhibited the upregulation of β-catenin, MMP-7, Drp1, and TGF-β1 expression, while preserving Tomm20 expression (Fig. 7f). Immunostaining further demonstrated that the levels of β-catenin, mtROS production, Drp1, and TGF-β1/α-SMA were suppressed, whereas E-cadherin was restored in mice subjected to early and late treatments (Fig. 7g; Additional file 1: Fig. S7a). These results suggested that cellular senescence and the β-catenin-Drp1-TGF-β1 pathway formed a reciprocal cycle to accelerate peritoneal fibrosis. Indeed, we found that TGF-β1 induced the activation of β-catenin in mesothelial cells (Additional file 1: Fig. S7b).

## Discussion

**Fig. 8:**
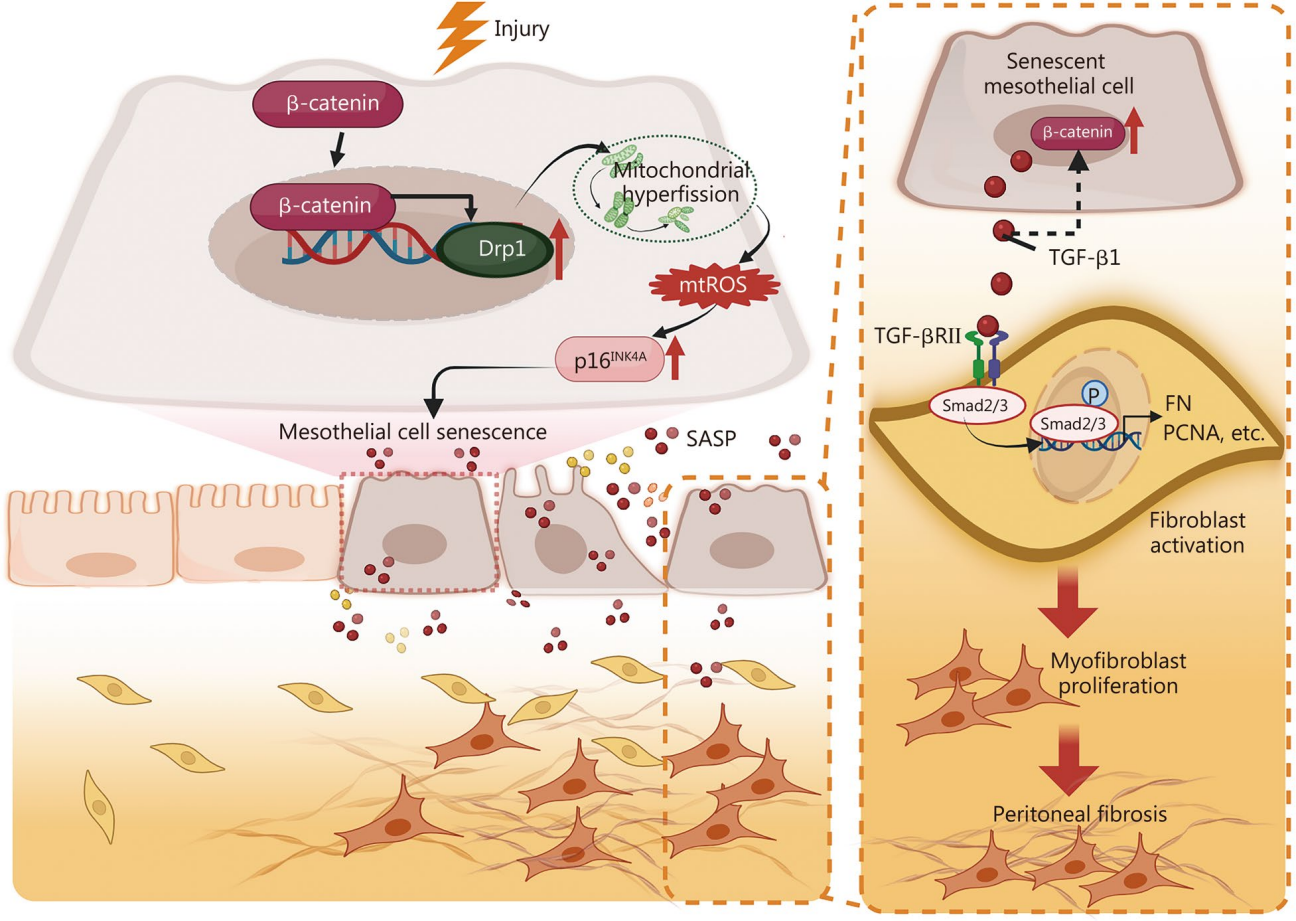
Schematic diagram. The schematic diagram illustrates the key role of β-catenin signaling in peritoneal fibrosis. As shown, upon injury, such as high glucose exposure, frequent mechanical stress, and chronic inflammation, peritoneal mesothelial cells would undergo cell fate transition to senescence. Increased β-catenin signaling plays a critical role in mediating this process. Through triggering Drp1-induced mitochondrial hyperfission, β-catenin induced excessive mtROS production to initiate p16^INK4A^-senescence pathways. Furthermore, senescent mesothelial cells release various SASP molecules to promote myofibroblast overactivation and matrix deposition. Terminally, peritoneal fibrosis will progress irreversibly, leading to PD failure. Hence, targeting β-catenin or cell senescence should be comprehensively considered in future peritoneal dialysis. Drp1 dynamin-related protein 1, mtROS mitochondrial reactive oxygen species, SASP senescence-associated secretory phenotype, TGF-β1 transforming growth factor-β1, TGF-βRII transforming growth factor-β receptor type II, PCNA proliferating cell nuclear antigen, ECM extracellular matrix

In this study, we reveal a new mechanism and implicate the potential therapeutic targets for treating peritoneal fibrosis. Upon long-term PD, β-catenin is activated to further induce Drp1-triggered mitochondrial hyperfission and mtROS overproduction in mesothelial cells. This leads to p16^INK4A^ activation and cellular senescence. Furthermore, senescent mesothelial cells produce TGF-β, a SASP molecule, to activate fibroblasts through the TGF-β/Smad pathway. All these cooperatively result in peritoneal fibrosis **(**Fig. 8**)**.

Previous study showed that mesothelial cells could undergo an aged phenotype [13]; however, the existence of mesothelial cell senescence in PD patients, as well as the underlying mechanisms, needs to be systematically investigated. We demonstrated that mesothelial cells undergo senescence transition under long-term PD and identified that β-catenin is associated with the process. Herein, we defined TGF-β as a key SASP molecule [39] to mediate the communication between mesothelial cells and fibroblasts. Several proofs support our findings. First, rigorous scRNA sequencing analysis showed mesothelial cells transition to senescence phenotype under long-term PD, accompanied with high production of TGF-β1 (Fig. 1). Second, in primary human mesothelial cells and peritoneum samples from PD patients, we found strong expression of p16^INK4A^ and other senescence markers, accompanied by β-catenin activation (Fig. 2). Third, gene ablation of β-catenin or pharmacological inhibition of β-catenin strongly resists mesothelial cell senescence and peritoneal fibrosis (Fig. 6; Additional file 1: Fig. S6). Finally, we found TGF-βRII and p-Smad, the receptor of TGF-β1 and its downstream effector, are activated in fibroblasts (Additional file 1: Fig. S5), suggesting the intercellular crosstalks by SASP molecules.

From various reports [19, 40], including our present study, we can conclude that mesothelial cells largely exhibit MMT phenotypes under long-term PD. Whereas, these MMT cells finally step forward into senescence fate (Fig. 1; Additional file 1: Fig. S1). Of note, these MMT cells possess some features similar to myofibroblasts, such as ECM production. For example, from the gene expression profile, we observed that Meso_1 (Inj_1) to Meso_3 (Inj_3) clusters could also highly produce collagens (Additional file 1: Fig. S1g). However, these cells are not true myofibroblasts because true myofibroblasts have strong proliferative abilities, while the mesothelial cells from long-term PD patients have lower expression of PCNA, a proliferative marker [41]. Hence, because of the superior capability of ECM production in myofibroblasts, we believe that myofibroblasts, the major cell type for fibrotic scarring in organ fibrosis [42], are the key cell type for ECM production in peritoneal fibrosis. Consistently, we found that under long-term PD, the collagen deposition area is primarily located in the submesothelial layer, the location of myofibroblasts (Fig. 1).

Drp1, a dynamin family member, splits mitochondrial tubules into fragments through binding the mitochondrial outer membrane and a larger oligomer assembly [43, 44]. Herein, we also observed dynamic mitochondrial changes in senescent mesothelial cells. We found that Drp1 was highly increased in mesothelial cells derived from long-term PD patients. We also confirmed that β-catenin upregulates *Drp1* mRNA and protein expression through transcription factor TCF4. This highly links mitochondrial dynamics with β-catenin signaling, implicating the multiple roles of β-catenin in regulating cellular senescence.

Senescent cells produce SASP factors to change the microenvironment and alter intercellular communication [45]. This study highly reveals that TGF-β1 facilitates the communication between senescent mesothelial cells and fibroblasts, which is controlled via β-catenin, underscoring the pivotal role of the β-catenin-Drp1-TGF-β1 axis in peritoneal fibrosis.

Cell senescence is different from other forms of cell cycle arrest, such as programmed cell death and terminally differentiated cells, by distinctive markers and morphological changes [46]. In this study, we found mesothelial cells undergo cell senescence transition from several lines of proofs, such as scRNA sequencing, senescence morphology, and markers identification, and the therapeutic response to senolytic agents. For the first time, the senolytic drugs dasatinib (D) and quercetin (Q) were administered to PD-associated fibrosis mice. These compounds have been shown to effectively eliminate senescent cells in various tissues [32, 47], but their efficacy in peritoneal fibrosis has not been evaluated till now. Remarkably, the administration of D + Q at two different time points, immediately or four weeks after PDF injection, successfully reduced senescent mesothelial cells and significantly attenuated fibroblast activation and peritoneal fibrosis progression. Furthermore, treatment with senolytic drugs could preserve mesothelial expression of E-cadherin, suggesting their certain therapeutic effects on preventing MMT. Besides clearing senescent cells, these senolytic drugs could also manifest certain anti-inflammatory effects, which may explain why they also prevent MMT. Of interest, early treatment (D + Q-ET) showed greater efficiencies in reducing senescence burden and resisting peritoneal fibrosis, compared to late treatment (D + Q-LT). Although the difference was not statistically significant, we still recommend applying the senolytic drugs in the early stage, because damage is irreversible in the late stage, and senescent cells would certainly affect the function of neighboring cells. Notably, we found no significant adverse effects in D + Q-treated mice compared to vehicle controls, supporting the relative safety of this treatment. Given that p16^INK4A^- and TGF-β1-positive cells were predominantly localized in the mesothelial layer, we believe that D + Q primarily target senescent mesothelial cells with minimal off-target effects. While further clinical translation remains a long-term goal, our findings provide a proof-of-concept for the therapeutic potential of senolytic agents in peritoneal fibrosis.

We further found D + Q treatment interrupted the β-catenin-Drp1-TGF-β1 axis, suggesting the notion of this reciprocal activation loop in peritoneal fibrosis, i.e., β-catenin-senescence-β-catenin loop. Although β-catenin was reported to be involved in cell senescence in other organs, such as the kidney and lung [28, 48], this study is the first to establish its critical role in mesothelial cell senescence. These findings further highlight the therapeutic potential of targeting β-catenin in age-related pathologies. Therefore, our work not only elucidates the underlying mechanisms of peritoneal fibrosis but also proposes a novel therapeutic strategy.

Several limitations existed in the current study. First, the numbers of peritoneal biopsies obtained from PD patients in this investigation were limited, and the clinical cohort of PD effluent samples was relatively small. Second, we observed that β-catenin induced various SASP factors, including TGF-β1, IL-8, and MCP-1. We believe that additional SASP factors remain to be identified, and their roles in peritoneal fibrosis warrant further investigation.

## Conclusions

In summary, our study reveals that senescent mesothelial cells were increased in the PD effluent as well as peritoneum biopsies of long-term PD patients, and more communication signals appeared between senescent mesothelial cells and fibroblasts. Our data clearly proved that mesothelial cell senescence strongly contributes to peritoneal fibrosis. Mechanistically, the β-catenin-Drp1-TGF-β1 axis is a key mediator for the initiation and progression of peritoneal fibrosis. Our findings supply new insights into understanding the mechanisms of peritoneal fibrosis, and importantly, provide novel therapeutic strategies to combat peritoneal fibrosis.

## Supplementary Information

**Additional file 1. **Methods.** Fig. S1** Clustering of peritoneal cells and re-clustering of mesothelial cells in scRNA-seq data.** Fig. S2** Long-term PD induces peritoneal mesothelial cell senescence accompanied by β-catenin and TGF-β signaling activation. **Fig. S3** Ectopic expression of β-catenin induces senescence and mitochondrial fission in mesothelial cells. **Fig. S4** Quantitative data for Fig. 5. **Fig. S5** Knockout of β-catenin disrupts the communications of mesothelial cells and fibroblasts, and alleviates peritoneal fibrosis. **Fig. S6** Pharmacological inhibition of β-catenin retards peritoneal mesothelial cell senescence and its communication with fibroblasts. **Fig. S7** TGF-β1 activates β-catenin in mesothelial cells. **Table S1** Clinical characteristics of the patients. **Table S2** Clinical characteristics of the PD patients. **Table S3** Clinical characteristics of 51 long-term PD patients. **Table S4** Genesets of fetal mesothelial cells hallmark, MMT, senescence, profibrosis, and ECM.

## Data Availability

All data associated with this study are present in the paper or additional file. Raw data of Western blotting are in data file. The sequencing data were uploaded to the SRA database under accession numbers PRJNA1249443 and PRJNA1255610.

## Abbreviations

ATP: Adenosine triphosphate
ChIP: Chromatin immunoprecipitation
CM: Conditioned medium
CCI: Cell–cell interaction
Drp1: Dynamin-related protein 1
ELISA: Enzyme-linked immunosorbent assay
ESRD: End-stage renal disease
ECM: Extracellular matrix
GO: Gene Ontology
GSEA: Gene set enrichment analysis
H3: Histone H3
IL-6: Interleukin-6
IL-8: Interleukin-8
KRT19: Cytokeratin 19
LEF: Lymphoid enhancer binding factor
MEF: Mouse embryonic fibroblasts
MMP: Mitochondrial membrane potential
MMP-7: Matrix metalloproteinase-7
MMT: Mesothelial-mesenchymal transition
mtDNA: Mitochondrial DNA
mtROS: Mitochondrial reactive oxygen species
MCP-1: Monocyte chemoattractant protein-1
MSLN: Mesothelin
PD: Peritoneal dialysis
PDF: Peritoneal dialysis fluid
PF: Peritoneal fibrosis
PCNA: Proliferating cell nuclear antigen
ROS: Reactive oxygen species
SASP: Senescence-associated secretory phenotype
SA-β-gal: Senescence-associated β-galactosidase
scRNA-seq: Single-cell RNA sequencing
TCF: T-cell factor
TGF-β1: Transforming growth factor-β1
TGF-βRII: TGF-β receptor II
TNF-α: Tumor necrosis factor α
TRRUST: Transcriptional Regulatory Relationships Unraveled by Sentence-based Text-mining
Tomm20: Mitochondrial import receptor subunit TOM20 homolog
UPK3B: Uroplakin 3B
WT1: Wilms tumor 1
γH2AX: Phosphorylated histone H2
