# Supplementary material for: β-catenin initiates peritoneal fibrosis by triggering mitochondrial fission-mediated mesothelial cell senescence fate transition
Source: Mil Med Res. 2025 Dec 1;12:83. doi: 10.1186/s40779-025-00669-1 (PMC12667144; doi:10.1186/s40779-025-00669-1)
Supplement: Supplementary file 1 — Additional file 1. Methods. Fig. S1 Clustering of peritoneal cells and re-clustering of mesothelial cells in scRNA-seq data. Fig. S2 Long-term PD induces peritoneal mesothelial cell senescence accompanied by β-catenin and TGF-β signaling activation. Fig. S3 Ectopic expression of β-catenin induces senescence and mitochondrial fission in mesothelial cells. Fig. S4 Quantitative data for Fig. 5. Fig. S5 Knockout of β-catenin disrupts the communications of mesothelial cells and fibroblasts, and alleviates peritoneal fibrosis. Fig. S6 Pharmacological inhibition of β-catenin retards peritoneal mesothelial cell senescence and its communication with fibroblasts. Fig. S7 TGF-β1 activates β-catenin in mesothelial cells. Table S1 Clinical characteristics of the patients. Table S2 Clinical characteristics of the PD patients. Table S3 Clinical characteristics of 51 long-term PD patients. Table S4 Genesets of fetal mesothelial cells hallmark, MMT, senescence, profibrosis, and ECM. [file 40779_2025_669_MOESM1_ESM.pdf]

Methods

Single-cell analysis

To test the cell fate of mesothelial cells, we extracted publicly available single-cell RNA sequencing (scRNA-seq) data (GSE130888), which was performed in human peritoneal cells from dialysate effluent. The samples were from both short- (n = 6) and long-term peritoneal dialysis (PD) patients (n = 4). The filtered matrix output was used to create a Seurat object for each sample, with genes expressed in more than 3 cells. Cells with at least 200 genes by using Seurat (version 4.2.3) were loaded and merged. The cells were excluded if the thresholds < 200 or > 6000 unique genes expressed, or > 30% of reads reflecting mitochondria. After quality control (QC) filters, a total of 76,721 cells, including 51,887 in the short-term PD group and 24,834 in the long-term PD group, were further analyzed. The NormalizeData function was used to normalize the raw read counts. A factor of 10,000 and taking $\log_2$-transformed values were scaled. The top 2000 highly variable features were detected by using the FindVariableFeatures function and then were used to calculate principal component analysis. To remove batch effect, Harmony (version 1.0) was used. The top 15 principal components were selected for unsupervised cell clustering and Uniform Manifold Approximation and Projection (UMAP). The FindClusters function with resolution = 0.25 was used to cluster cells for clustering. Differential marker gene expression analysis was used for screening each cluster using the "FindAllMarkers" function based on the following parameters: test.use = "Wilcox", logfc.threshold = 0.25 and adjusted P-value < 0.05. According to canonical markers of specific cell types and referring to CellMarker 2.0, the 12 clusters were identified and corresponded to mesothelial cells, macrophages, T cells, monocytes, NK/T cells, dendritic cells, proliferating cells, B cells, plamablasts, and neutrophils.

For re-analysis of the mesothelial cells, we extract the mesothelial cluster and process it through the procedures of harmony integration, Principal Component Analysis (PCA), and clustering. Cells were then re-clustered into 6 clusters with a resolution = 0.25. To annotate these subtypes, Differential gene expression (DGE) analysis of each cluster was performed: subcluster 2 and 6 showed high expression levels of marker genes associated with immune cells; therefore, they were considered likely to be contaminated immune cells; subcluster 1 exhibited high expression of fibrosis-related genes, hence it was considered potentially fibroblast-like cells. To identify the remaining subclusters of mesothelial cells, the AUCell package was employed to assess the cell state scores. The Monocle2 R package was applied to find the cell-state transitions. AUCell was processed with the pre-defined gene

sets of fetal mesothelial cells hallmark, EMT, senescence, and profibrosis (**Additional file 1: Table S3**). Using the FindAllMarkers function, pseudotime analysis was performed. Top 100 differential expression genes of each cluster with *Q*-value < 0.01 were used to construct a cell trajectory through the setOrderingFilter function procedure. According to the result of AUCell and cell trajectory analysis, subclusters 4 (Meso_0) were identified as healthy mesothelial, while the other 3 were considered as injury states. The Plot1cell package was applied to reveal the cellular senescence gene regulation in different states. For pathway analysis between long- and short-term PD groups in each subcluster, Gene Set Variation Analysis (GSVA) was used.

For further analysis of the injury state 3 mesothelial cells, the differentially expressed genes (min.pct = 0.1, *P*-value < 0.05, and avg_log_2 fold change ≥ 0.25) were analyzed using the FindMarkers function in Seurat, and then gene ontology-based pathway analysis and Transcriptional Regulatory Relationships Unraveled by Sentence-based Text-mining (TRRUST) enrichment were performed on Metascape (http://metascape.org). Gene Set Enrichment Analysis (GSEA) of the injury state 3 cell type between 2 groups was performed with "GOBP_MITOCHONDRIAL_FISSION" and "PID_BETA_CATENIN_NUC_PATHWAY" datasets, based on the Molecular Signatures Database of the GSEA web interface.

For cell-cell interaction analysis, the CellChat R package was used, depending on the ligand-receptor interaction in mesothelial subtypes. Normalized gene expression in the matrix and major subclusters of long- and short-term PD groups acted as input for CellChat. The human-specific CellChat DB was used with secreted signaling interaction, and cell communication was filtered out with min.cell = 5. Communication probabilities were calculated using 1000 permutations (nBoot = 1000), and significant interactions (*P* < 0.05) were retained for downstream pattern analysis. The functions mergeCellChat and compareInteractions were adopted to analyze different numbers of pairs.

Primary antibodies in Western blotting analysis

Primary antibodies used in Western blotting analyses included fibronectin (rabbit polyclonal; F3648; Sigma, USA; 1:5000), collagen type alpha 1 chain (COL1A1; rabbit polyclonal; BA0325; Boster Biotechnology, China; 1:1000), human/mouse E-cadherin (goat polyclonal; AF748; R&D Systems, USA; 1:1000), α-smooth muscle actin (α-SMA; mouse monoclonal; ab7817; Abcam, USA; 1:3000), TOMM20 (rabbit monoclonal; ab186735; Abcam, USA; 1:1000), dynamin-related protein 1 (Drp1) (rabbit monoclonal; ab184247; Abcam, USA; 1:1000), $p16^{INK4A}$ (mouse monoclonal; sc-1661; Santa

Cruz Biotechnology, USA; 1:1000), phosphorylated histone H2AX (γH2AX; rabbit monoclonal; A11412; Abclonal, China; 1:1000), CDKN1A/p21 (rabbt polyclonal; A2691; Abclonal, China; 1:1000), p53 (mouse monoclonal; sc-126; Santa Cruz Biotechnology, USA; 1:1000), β-catenin (mouse monoclonal; 610154; BD Biosciences, USA; 1:1000), non-phospho (active) β-catenin (rabbit monoclonal; 19807; Cell Signaling Technology, USA; 1:1000), matrix metalloproteinase-7 (MMP-7) (rabbit polyclonal; GTX57231; GeneTex, USA; 1:1000), proliferating cell nuclear antigen (PCNA) (mouse monoclonal; ab29; Abcam, USA; 1:3000), transforming growth factor-β1 (TGF-β1) (mouse monoclonal; sc-130348; Santa Cruz Biotechnology, USA; 1:1000), TGF-β receptor 2/TGFBR2 (mouse monoclonal; sc-17791; Santa Cruz Biotechnology, USA; 1:1000), phospho-Smad2 (pSmad2; 3108S; rabbit monoclonal; Cell Signaling Technology, USA; 1:1000), phospho-Smad3 (pSmad3; rabbit monoclonal; AP0727; ABclonal, China; 1:1000), Flag (mouse monoclonal; M30971-2; Boster Biological Technology, China; 1:1000), GAPDH (mouse monoclonal; RM2002; Ray Antibody Biotech, China; 1:5000), and α-tubulin (mouse monoclonal; RM2007; Ray Antibody Biotech, China; 1:5000). The signals were detected by X-ray film and quantified by ImageJ software.

Cell treatment

Recombinant human TGF-β1 protein (10 ng/ml) (R&D Systems; USA) was used to treat HMrSV5 cells for 48 h. In some experiments, HMrSV5 cells were transfected with β-catenin plasmid or empty vector for 6 h, followed by incubation with serum-free medium for 24 h. The supernatant was collected and used as conditioned medium (CM). Mouse embryonic fibroblasts (MEFs) were subsequently treated with 30% CM for 24 h.

In separate experiments, MEFs were preincubated with a neutralizing antibody against TGF-β1 receptor II (AF241-NA; R&D Systems, USA; 5 μg/ml) for 1 h prior to treatment with β-catenin-CM for 24 h. In additional experiments, MEFs were treated with recombinant TGF-β1 protein at a low concentration of 250 pg/ml for 48 h.

Enzyme-linked immunosorbent assay (ELISA)

Levels of MMP-7 and TGF-β1 in PD effluent were quantified using ELISA following standardized protocols; the absorbance at 450 nm was recorded using a SYNERGY HTX multi-mode reader (BioTek, USA). Human MMP-7 (EH10465; Wellbio, China) and TGF-β1 Assay Kits (EH10357; Wellbio, China) were purchased from Wellbio Technology. The levels of TGF-β1, tumor necrosis factor-α (TNF-α), interleukin (IL)-6, IL-8, and monocyte chemoattractant protein-1 (MCP-1) in CM

were measured by human TGF-β1 (EH10465; Wellbio, China), TNF-α (EH10497; Wellbio, China), IL-6 (EH10293; Wellbio, China), IL-8 (EH102961159; Wellbio, China), and MCP-1(EH10335; Wellbio, China) ELISA Kits, respectively.

SA-β-gal staining

SA-β-gal staining was performed on frozen sections of the parietal peritoneum (6 μm thickness) and cell slides with a commercial kit (C0602; Beyotime, China) at 37 °C for 24 to 48 h according to the manufacturer's protocol.

Quantitative real-time PCR (qRT-PCR)

Total RNA was extracted with TRIzol reagent (Life Technologies, Grand Island, NY, USA). qRT-PCR was conducted according to the standard protocol. Sequences of the primers used as follows: *β-catenin* forward 5′-GCCCTGGTGAAAATGCTTGG-3′ and reverse 5′-CGCACTGCCATTTTAGCTCC-3′, *DNM1L* (encoding Drp1) forward 5′-CTGCCTCAAATCGTCGTAGTG-3′ and reverse 5′-GAGGTCTCCGGGTGACAATTC-3′, *β-actin* forward 5′-CTCACCATGGATGATGATATCGC-3' and reverse 5′-AGGAATCCTTCTGACCCATGC-3′. The Ct values were normalized to β-actin and the relative gene expression was measured by $2^{-\Delta\Delta Ct}$.

Measurement of adenosine triphosphate (ATP) levels

Contents of ATP were assessed using an enhanced ATP assay kit (S0027; Beyotime, China) following the manufacturer's protocol.

Transmission electron microscopy (TEM)

Primary human mesothelial cells and HMrSV5 cells were collected and fixed with 1.25% glutaraldehyde in 0.1 mmol/L phosphate buffer. The ultrastructure of mitochondria was observed by transmission electron microscope (JEM-400 Plus, JEOL, Japan).

5-Ethynyl-2'-deoxyuridine (EdU)-based cell proliferation assay

MEFs' cell proliferation was assessed by a Click-iT EdU assay kit (C10337; Thermo Fisher Scientific, USA) following the supplier's guidelines. Briefly, MEFs were plated and incubated with 10 μmol/L EdU for 2 h before fixation and permeabilization. Subsequently, MEFs were treated with EdU reaction solution, followed by nuclear staining with Hoechst (C1018; Beyotime, China). The numbers of EdU-positive MEF cells were counted in 10 random views and normalized to the nuclei.

Detection of mitochondrial reactive oxygen species (mtROS)

After treatment, the HMrSV5 cells were incubated with 5 μmol/L MitoSOX dye (M36008, Thermo Fisher Scientific, USA) at 37 °C for 10 min. Cellular ROS generation was evaluated through flow cytometric analysis using an emission wavelength of 580 nm, and the data were analysed using FlowJo software. Mitochondrial ROS levels in both parietal peritoneum and treated cells were observed by confocal microscopy after MitoSOX Red (M36008, Thermo Fisher Scientific, USA) staining.

Detection of mitochondrial function

JC-1 fluorescent probe (40705ES03, Yeasen Biotechnology, China) was employed to evaluate mitochondrial membrane potential (MMP), with the red/green fluorescence ratio (JC-1 aggregates/monomers) serving as the MMP indicator. Mitochondrial morphology was examined using MitoTracker Deep Red (M22426, Thermo Fisher Scientific, USA) according to established protocols.

Primary antibodies in immunofluorescence staining

Primary antibodies used in immunofluorescence staining included β-catenin (610154; BD Biosciences, USA; 1:100), $p16^{INK4A}$ (10883-1-AP; Proteintech, USA; 1:50), uroplakin 3B (UPK3B) (ab237778; Abcam, USA; 1:50; ab35546; Absci; USA; 1:50), hector battifora mesothelial cell-1 (HBME1) (sc-59307; Santa Cruz Biotechnology, USA; 1:50), wilms tumor protein 1 (WT1) (BM4216; Boster Biological Technology, China; 1:50), active-β-catenin (19807; Cell Signaling Technology, USA; 1:50), Drp1 (ab184247; Abcam, USA; 1:50), TGF-β1 (sc-130348; Santa Cruz Biotechnology, USA; 1:50), E-cadherin (AF748; R&D System, USA; 1:100), transforming growth factor-β receptor type II (TGF-βRII) (AF-241-NA; R&D System, USA; 1:50), fibroblast-specific protein 1(FSP1) (ab218512; Abcam, USA; 1:50), pSmad3 (AP0727; ABclonal, China; 1:50), fibronectin (F3648; Sigma, USA; 1:200), and α-SMA (14395-1-AP; Proteintech, USA;1:100).

RNA sequencing (RNA-seq)

Briefly, HMrSV5 cells were transfected with pcDNA3 or β-catenin expression plasmid (pFlag-β-catenin) (n = 2), and their total RNA was collected after 2 days of transfection. Subsequent RNA-seq and data analysis were performed through Gene Denovo company (Guangzhou, China). MEFs were treated with 30% HMrSV5 cell-CM for 24 h (n = 3), and total RNA was extracted using the above method; the RNA-seq analysis was supported by Biotechnology Corporation (Shanghai, China).

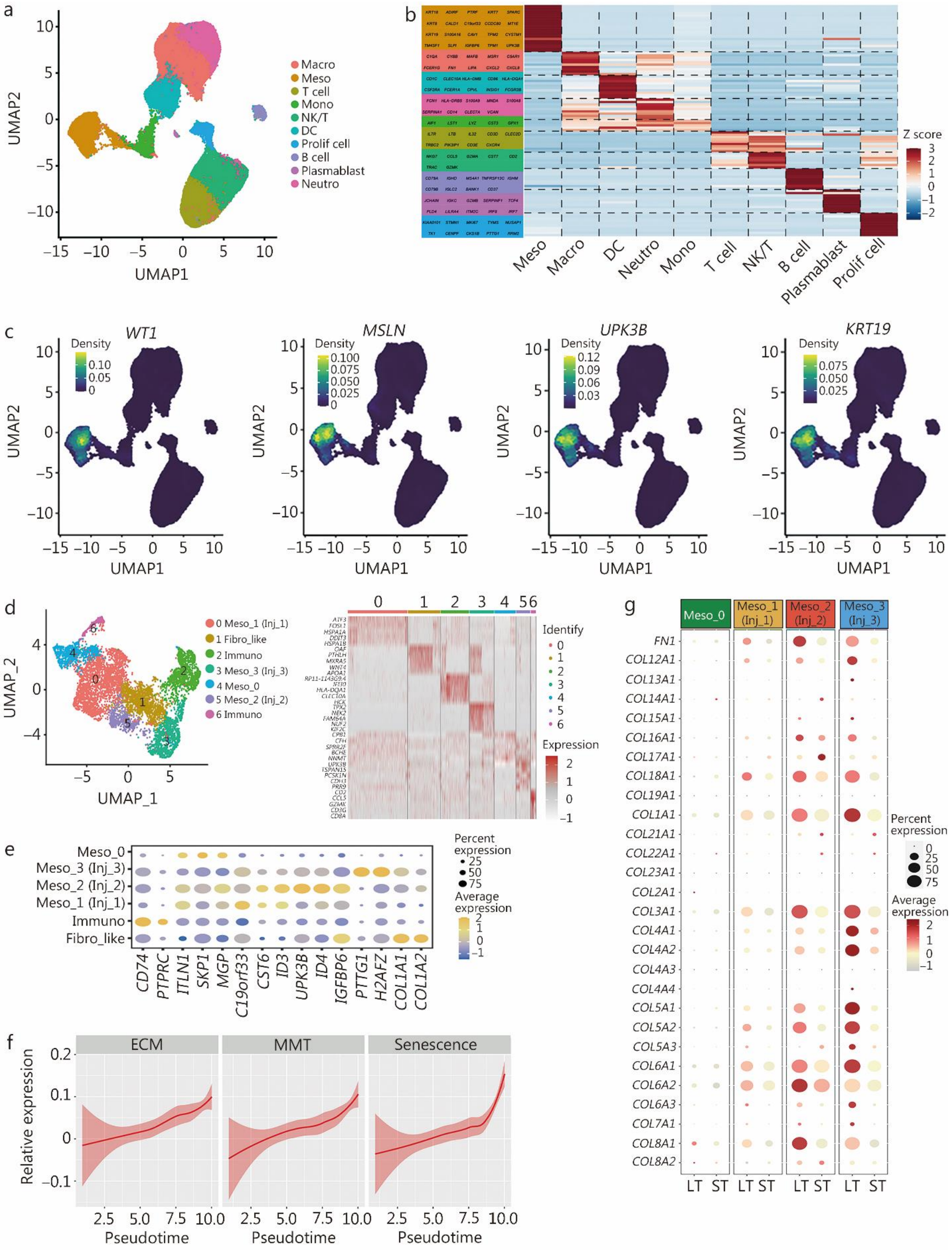

Fig. S1 Clustering of peritoneal cells and re-clustering of mesothelial cells in scRNA-seq data. **a** UMAP visualization showing unsupervised scRNA-seq clustering of human peritoneal cells from the effluent of long- (n = 4) or short-term PD patients (n = 6), revealing 10 distinct cell types. **b** Heatmap showing the top gene expressions of each cell type, and the scaled color bar represents the Z score. **c**

Feature plot showing marker genes of mesothelial cells. The scaled color bar represented the density of gene expression. **d** UMAP visualization showing unsupervised re-clustering of mesothelial cells, a heatmap showing the top 5 gene expression of each subcluster of mesothelial cells. **e** Dot plot displaying gene expression patterns of the reassigned cell types. **f** Histogram showing the expression scores for genes related to ECM, MMT, and senescence in the Meso_3 (Inj_3) cluster, along with the pseudotime. **g** The bubble plots show gene expression of ECM genes in the subclusters of mesothelial cells. Macro macrophage, Meso mesothelial cells, Mono monocyte, NK/T natural killer/T cells, DC dendritic cells, Prolif. cell proliferating cells, Neutro neutrophil, Fibro_like fibroblast-like cells, immuno immunocompetent cells, WT1 wilms tumor protein 1, MSLN mesothelin, UPK3B uroplakin 3B, KRT19 cytokeratin-19, ECM extracellular matrix, MMT mesothelial-mesenchymal transition, ST short-term, LT long-term, scRNA-seq single-cell RNA sequencing, UMAP Uniform Manifold Approximation and Projection

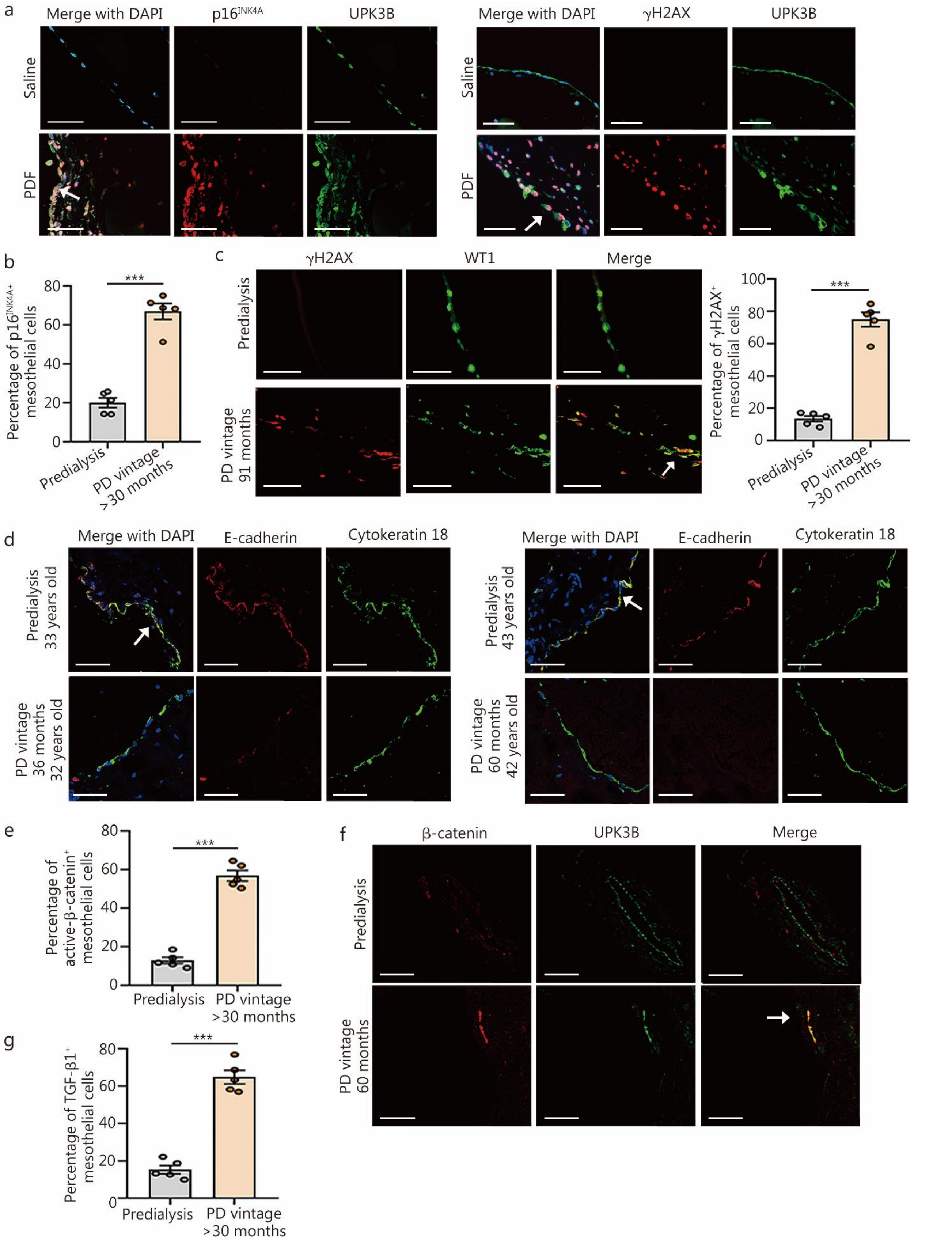

Fig. S2 Long-term PD induces peritoneal mesothelial cell senescence accompanied by β-catenin and TGF-β signaling activation. **a** Three-color immunofluorescence staining for DAPI, UPK3B, and p16^{INK4A} or γH2AX in the peritoneum of mice treated with saline or PDF. Scale bar = 50 μm. White

arrows indicate positive staining. **b** Representative images are shown in Fig. 1j. The proportion of $p16^{INK4A}$-positive mesothelial cells was increased in the peritoneums of patients undergoing long-term PD. The percentages of $p16^{INK4A+}$ mesothelial cells were counted in 5 random views of the peritoneum slide from one patient. Mesothelial cells are identified by a mesothelial cell marker. $^{***}P < 0.001$, by unpaired, two-tailed Student's *t*-test ($n = 5$). **c** Representative micrographs and quantification analysis of colocalization of γH2AX and WT1 in 2 groups. The white arrow indicates positive staining. Scale bar = 50 μm. $^{***}P < 0.001$, by unpaired, two-tailed Student's *t*-test ($n = 5$). **d** Three-color immunofluorescence staining for DAPI (blue), E-cadherin (red), and mesothelial cell marker cytokeratin 18 (green) in human peritoneums from pre-dialysis patients and long-term PD patients. Scale bar = 50 μm. White arrows indicate positive staining. **e** Representative images are shown in Fig. 2f, and quantitative data show the percentage of co-staining of active-β-catenin and UPK3B in mesothelial cells from human peritoneums. $^{***}P < 0.001$, by unpaired, two-tailed Student's *t*-test ($n = 5$). **f** Colocalization of β-catenin and UPK3B in human peritoneums. White arrows indicate positive staining. Scale bar = 25 μm. **g** Representative images are shown in Fig. 2g, and quantitative data show the percentage of co-staining of TGF-β1 and UPK3B in mesothelial cells from human peritoneums. $^{***}P < 0.001$, by unpaired, two-tailed Student's *t*-test ($n = 5$). PDF peritoneal dialysis fluid, UPK3B uroplakin 3B, γH2AX phosphorylated histone H2AX, WT1 wilms tumor protein 1, DAPI 4′,6-diamidino-2-phenylindole, WT1 wilms tumor protein 1, PD peritoneal daialysis, TGF-β1 transforming growth factor-β1

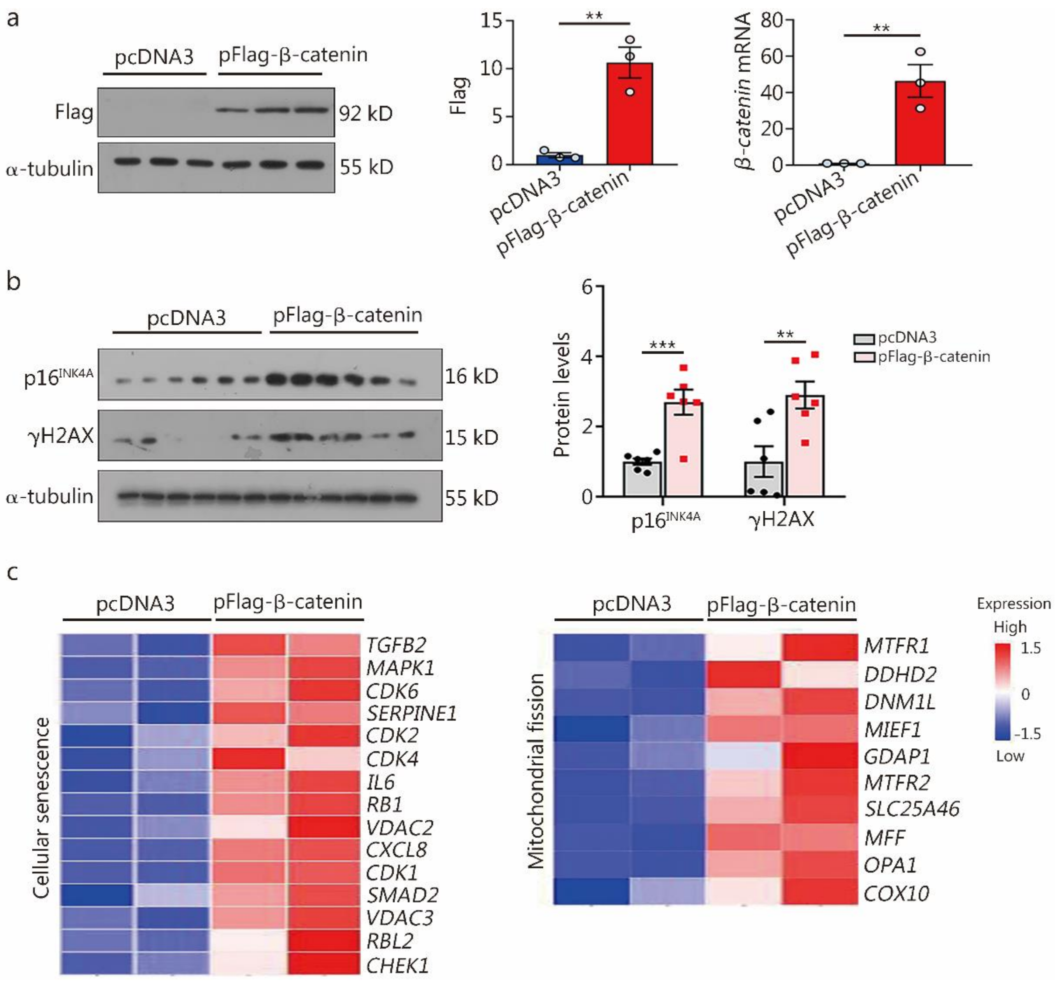

Fig. S3 Ectopic expression of β-catenin induces senescence and mitochondrial fission in mesothelial cells. **a** Western blotting analyses and qPCR results confirmed the transfection efficiency of the β-catenin expression plasmid. $^{**}P < 0.01$, by unpaired, two-tailed Student's *t*-test ($n = 3$). **b** Western blotting of p16^{INK4A} and γH2AX in 2 groups. Quantifications are shown. $^{**}P < 0.01$, $^{***}P < 0.001$, by unpaired, two-tailed Student's *t*-test ($n = 6$). **c** Heatmap showing the core gene expression of cellular senescence and mitochondrial fission in HMrSV5 cells transfected with pFlag-β-catenin compared to those transfected with pcDNA3. The color gradient refers to the scale expression level, standardizing gene expression using the scale function. The color gradient from dark blue to dark red refers to the gene expression level from low to high. $n = 2$. γH2AX phosphorylated histone H2AX

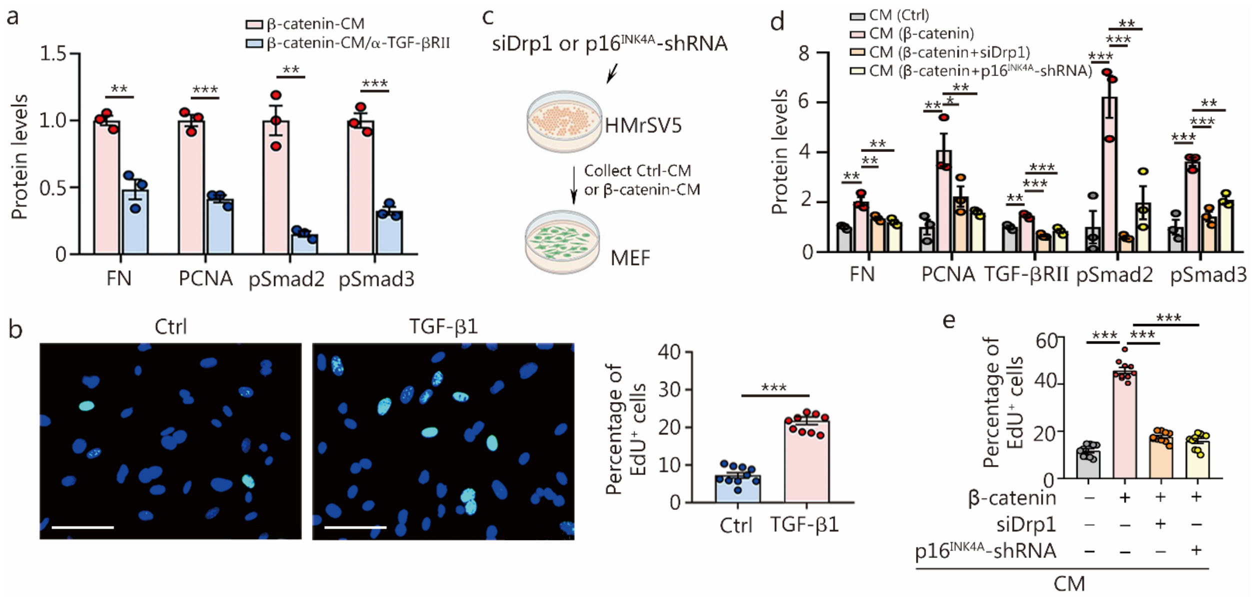

Fig. S4 Quantitative data for Fig. 5. **a** Quantitative data of Fig. 5j. $^{**}P < 0.01$, $^{***}P < 0.001$, by unpaired, two-tailed Student's *t*-test (n = 3). **b** EdU staining and its quantification in MEFs. $^{***}P < 0.001$, by unpaired, two-tailed Student's *t*-test (n = 10). **c** Experimental procedures. HMrSV5 cells were co-transfected with the β-catenin plasmid and siDrp1, or p16^{INK4A}-shRNA, for 6 h. After that, the serum-free medium was incubated for 24 h. The supernatant was then harvested for CM to stimulate MEFs. **d** Quantitative data for Fig. 5k. $^{*}P < 0.05$, $^{**}P < 0.01$, $^{***}P < 0.001$, by one-way ANOVA followed by the Least Significant Difference test (n =3). **e** EdU-positive cells were counted, and the representative images are shown in Fig. 5l. $^{***}P < 0.001$, by one-way ANOVA followed by the Least Significant Difference test (n = 10). FN fibronectin, PCNA proliferating cell nuclear antigen, MEF mouse embryonic fibroblast, siDrp1 dynamin-related protein 1 small interfering RNA, p16^{INK4A} shRNA p16^{INK4A} short hairpin RNA, CM conditioned medium, TGF-βRII transforming growth factor-β receptor type II, siDrp1 dynamin-related protein 1 small interfering RNA, TGF-β1 transforming growth factor-β1, pSmad2 phosphorylated Smad 2, pSmad3 phosphorylated Smad 3, Edu 5-Ethynyl-2'-deoxyuridine

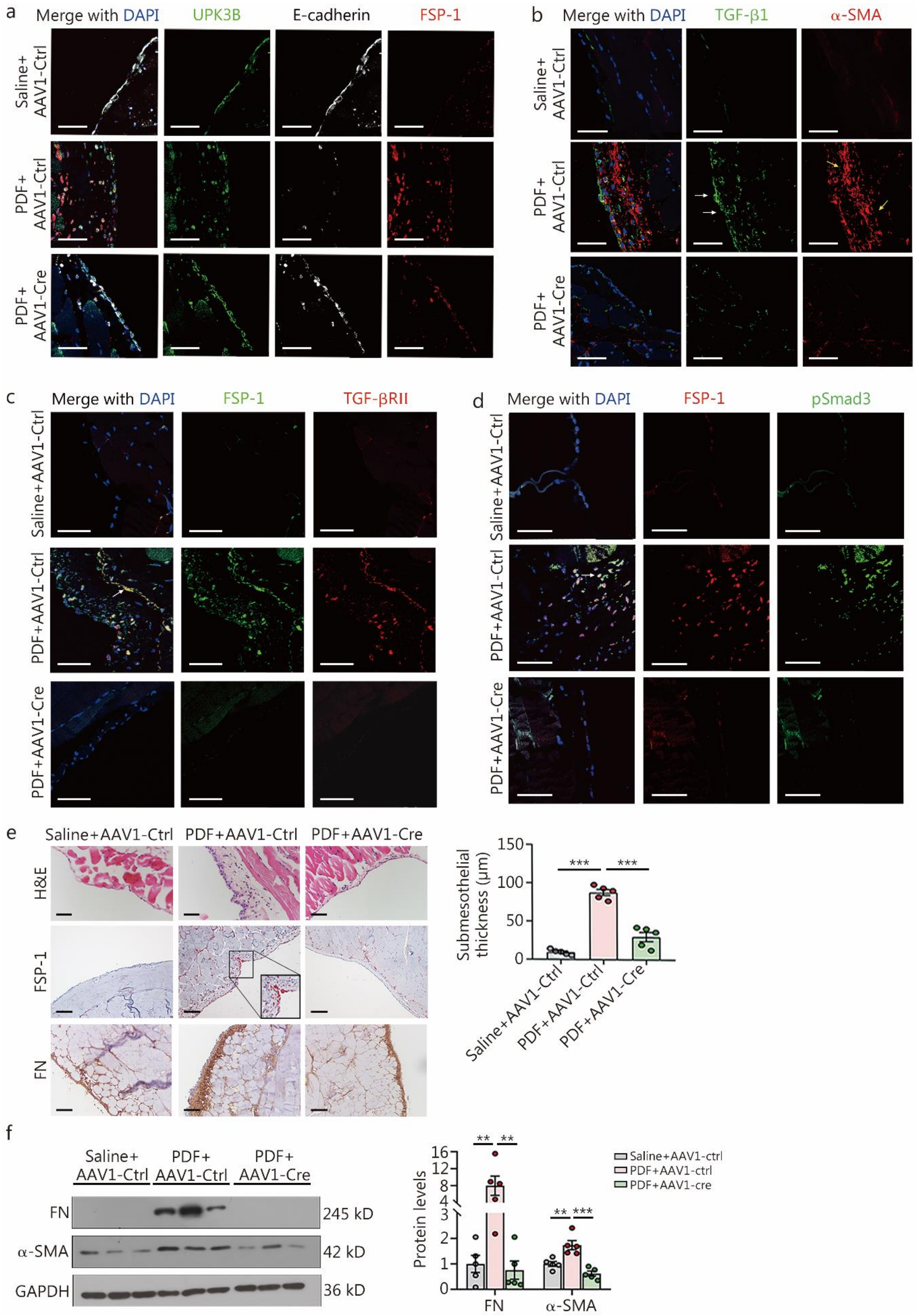

Fig. S5 Knockout of *β-catenin* disrupts the communications of mesothelial cells and fibroblasts, and alleviates peritoneal fibrosis. **a** Four-color immunofluorescence staining for DAPI (blue), UPK3B (green), E-cadherin (white), and FSP-1 (red) in parietal peritoneal sections from 3 groups. Scale bar =

50 μm. **b** Three-color staining for DAPI (blue), TGF-β1 (green), and α-SMA (red) in parietal peritoneal sections. White arrows indicate TGF-β1 positive staining, yellow arrows indicate α-SMA positive staining. Scale bar = 50 μm. **c** Three-color staining for DAPI (blue), FSP-1 (green), and TGF-βRII (red) in the parietal peritoneum. FSP-1/TGF-βRII double-positive cells are shown in PDF + AAV1-Ctrl-treated mice, but knockout of *β-catenin* reduces these double-positive cells. White arrows indicate FSP-1/TGF-βRII double-positive fibroblasts. Scale bar = 50 μm. **d** Three-color staining for DAPI (blue), FSP-1 (red), and pSmad3 (green) in parietal peritoneal sections from three groups. White arrows indicate FSP-1/pSmad3 double-positive fibroblasts. Scale bar = 50 μm. **e** H&E staining, immunohistochemical staining of FSP-1 and FN. For H&E staining, scale bar = 50 μm; for FSP-1 and FN staining, scale bar = 100 μm. Quantitation of peritoneal thickness in different groups, $^{***}P < 0.001$, by one-way ANOVA followed by Dunnett's T3 procedure test (n = 5). **f** Western blotting and quantitative data of FN and α-SMA. $^{**}P < 0.01$, $^{***}P < 0.001$, by one-way ANOVA followed by the Least Significant Difference test (n = 5). UPK3B uroplakin 3B, PDF peritoneal dialysis fluid, FN fibronectin, α-SMA α-smooth muscle actin, TGF-β1 transforming growth factor-β1, FSP-1 fibroblast-specific protein 1, TGF-βRII transforming growth factor-β receptor type II, H&E hematoxylin and eosin, DAPI 4′,6-diamidino-2-phenylindole, AAV1-Cre adeno-associated virus serotype 1-carried the Cre recombinase

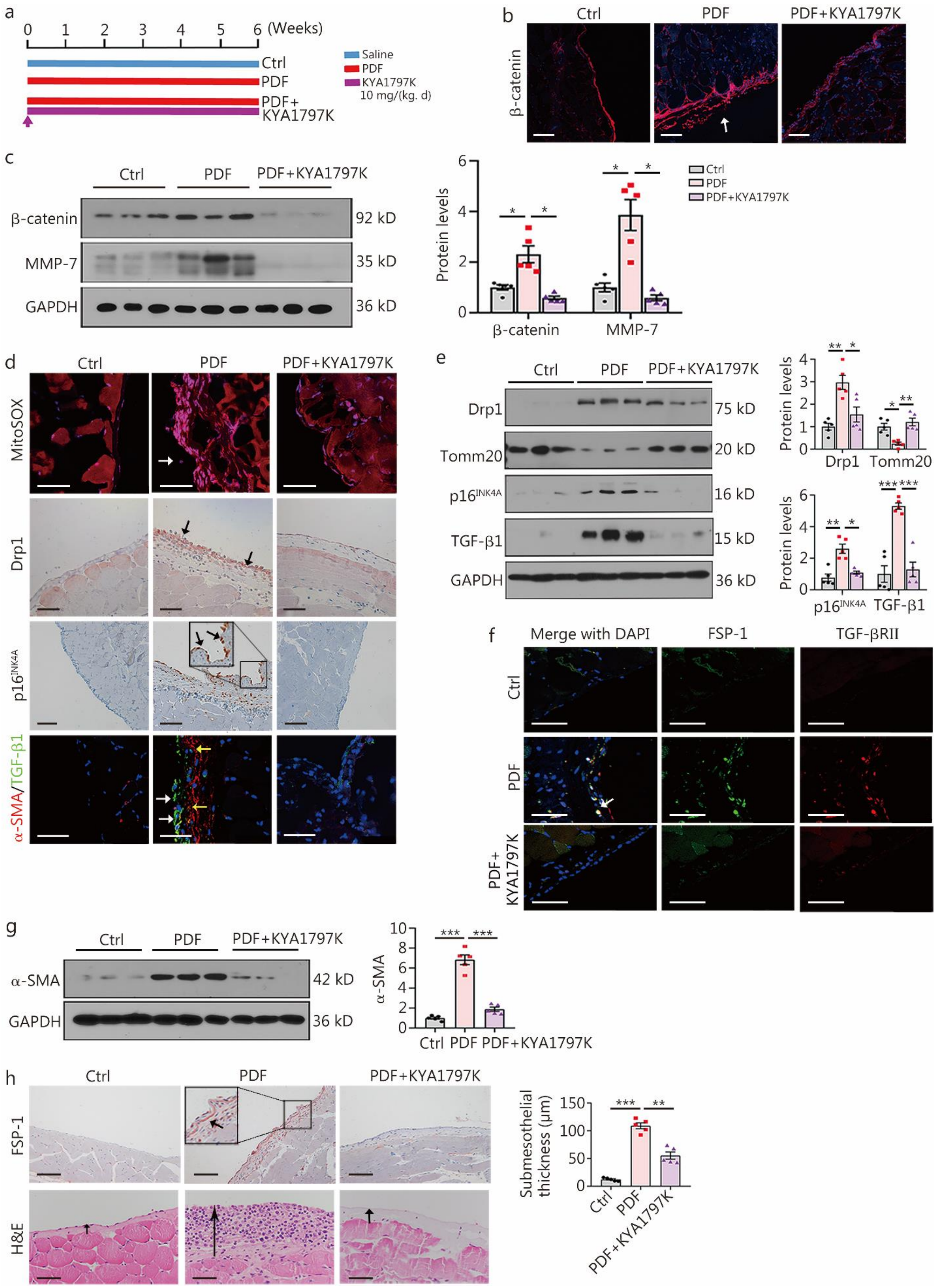

Fig. S6 Pharmacological inhibition of β-catenin retards peritoneal mesothelial cell senescence and its communication with fibroblasts. **a** Experimental design. KYA1797K was administered daily starting from the first day of PDF administration. **b** β-catenin staining in 3 groups. Arrow indicates positive

staining. Scale bar = 100 μm. **c** Western blotting and quantitative data of β-catenin and MMP-7. $^{*}P < 0.05$, by one-way ANOVA followed by Dunnett's T3 procedure test ($n = 5$). **d** The staining of MitoSOX, Drp1, p16^{INK4A} and α-SMA/TGF-β1 in the peritoneum from different groups. Arrows indicate positive staining. For MitoSOX, Drp1, and α-SMA/TGF-β1 staining, scale bar = 50 μm; for p16^{INK4A} staining, scale bar = 100 μm. **e** Western blotting and quantitative data of Drp1, Tomm20, p16^{INK4A} and TGF-β1. $^{*}P < 0.05$, $^{**}P < 0.01$, $^{***}P < 0.001$, for Drp1, Tomm20, and p16^{INK4A}, by one-way ANOVA followed by Dunnett's T3 procedure test; for TGF-β1, by one-way ANOVA followed by the Least Significant Difference test ($n = 5$). **f** Three-color staining for DAPI (blue), FSP-1 (green), and TGF-βRII (red). The white arrow indicates positive staining. Scale bar = 50 μm. **g** Western blotting analyses and quantitative data of α-SMA. $^{***}P < 0.001$, by one-way ANOVA followed by Dunnett's T3 procedure test ($n = 5$). **h** The staining for FSP-1 and H&E. Arrows indicate positive staining. For FSP-1 staining, scale bar = 100 μm; for H&E staining, scale bar = 50 μm. Peritoneal thickness was quantified. $^{**}P < 0.01$, $^{***}P < 0.001$, by one-way ANOVA followed by Dunnett's T3 procedure test ($n = 5$). PDF peritoneal dialysis fluid, DAPI 4′,6-diamidino-2-phenylindole, MMP-7 metalloproteinase-7, Drp1 dynamin-related protein 1, α-SMA α-smooth muscle actin, TGF-β1 transforming growth factor-β1, Tomm20 mitochondrial import receptor subunit TOM20 homolog, FSP-1 fibroblast-specific protein 1, H&E hematoxylin and eosin, MitoSOX mitochondrial superoxide indicator

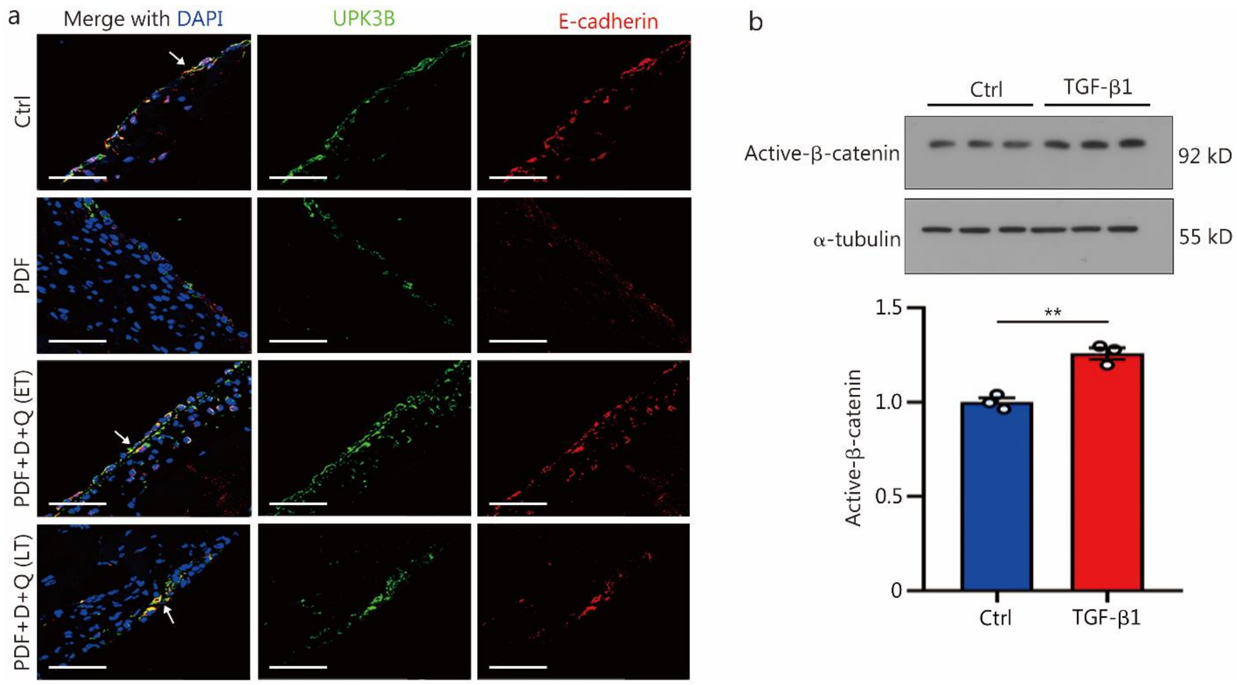

Fig. S7 TGF-β1 activates β-catenin in mesothelial cells. **a** Three-color staining for DAPI (blue), UPK3B (green), and E-cadherin (red) in parietal peritoneal sections from 4 groups. E-cadherin/UPK3B double-positive mesothelial cells were indicated by white arrows. Scale bar = 50 μm. **b** Western blotting and quantitative data of active-β-catenin, $^{**}P < 0.01$, by unpaired, two-tailed Student's *t*-test ($n = 3$). UPK3B uroplakin 3B, TGF-β1 transforming growth factor-β1, D + Q dasatinib plus quercetin, ET early treatment, LT late treatment, DAPI 4′,6-diamidino-2-phenylindole

Table S1 Clinical characteristics of the patients

No.	Gender	Age (years)	Time on PD (months)	Glucose concentration (%)	Causes of ESRD	Diabetes mellitus	HBP	BMI (kg/m^2)	Operation	History of peritonitis
1	Men	33	0	-	Unknown cause	No	Yes	21.9	PD catheter implantation	No
2	Men	43	0	-	DN	Yes	Yes	27.6	PD catheter implantation	No
3	Men	36	0	-	Unknown cause	No	Yes	18.2	PD catheter implantation	No
4	Female	38	0	-	Hypertention	No	Yes	25.5	PD catheter implantation	No
5	Men	48	0	-	DN	Yes	Yes	21.8	PD catheter implantation	No
6	Men	39	66	2.0	Unknown cause	No	Yes	39.5	PD catheter exit	No
7	Female	42	60	2.5	DN	Yes	Yes	22.9	PD catheter exit	Yes
8	Female	32	36	1.5	Unknown cause	No	Yes	22.4	PD catheter exit	No
9	Female	46	33	1.5	DN	Yes	Yes	20.4	PD catheter exit	Yes
10	Female	40	91	2.0	Unknown cause	No	Yes	22.3	PD catheter exit	No

The information of patients who provided parietal peritoneum samples used in Fig. 1j, Fig. 2f, g, h, Fig. 3g, and Fig. S2. "-" indicates not applicable, *DN* diabetic nephropathy, *HBP* high blood pressure, *PD* peritoneal dialysis, *ERSD* end-stage renal disease, *BMI* body mass index

Table S2 Clinical characteristics of the PD patient

No.	Gender	Age (years)	Time on PD (months)	Glucose concentration (%)	Causes of ESRD	Diabetes mellitus	HBP	BMI (kg/m^2)	PET	History of peritonitis
1	Female	38	1	1.5	Unknown cause	No	Yes	17.6	0.75 (high -average)	No
2	Men	35	3	1.5	DN	Yes	Yes	17.2	0.75 (high-average)	No
3	Female	47	2	1.5	Unknown cause	No	No	19.8	0.65 (high-average)	No
4	Men	28	3	1.5	FSGS	No	Yes	21.8	0.62 (low-average)	No
5	Femele	42	3	1.5	Unknown cause	No	Yes	27.8	0.75 (high-average)	No
6	Men	53	2	1.5	Unknown cause	No	No	24.5	0.62 (low-average)	No
7	Men	53	101	1.5	Unknown cause	No	Yes	24.4	0.75 (high-average)	No
8	Female	27	97	1.5	IgA N	No	Yes	27.2	0.50 (low-average)	No
9	Men	28	61	1.5	Unknown cause e	No	Yes	22.8	0.75 (high-average)	No
10	Men	42	146	1.5	IgA N	No	Yes	19.9	0.63 (high-average)	No
11	Female	54	64	2.0	IgA N	N0	Yes	22.2	0.83 (high-average)	No
12	Female	38	150	2.0	Unknown cause	No	Yes	16.0	0.80 (high-average)	No

The information of patients who provided dialysate effluent to isolate primary mesothelial cells used in Fig. 1e-i, Fig. 2d, e and Fig. 3d-f. *DN* diabetic nephropathy, *HBP* high blood pressure, *FSGS* focal segmental glomerulosclerosis, *IgA N* IgA nephropathy, *PD* peritoneal dialysis, *ERSD* end-stage renal disease, *BMI* body mass index, *PET* peritoneal equilibration test

Table S3 Clinical characteristics of 51 long-term PD patients

No.	Gender	Age (years)	PD vintage (months)	Dialysate MMP-7 (pg/ml)	Dialysate TGF-β1 (pg/ml)	Causes of ESRD	Diabetes mellitus	HBP	BMI (kg/m^2)	Glucose concentration (%)	PET
1	Female	18	62	53.79	144.10	MN	No	Yes	20.1	1.5	0.50 (low-average)
2	Female	25	74	25.21	120.93	Unknown cause	No	Yes	16.2	1.5	0.70 (high-average)
3	Female	25	43	109.68	75.85	Hypertention	No	Yes	17.9	1.5	0.59 (low-average)
4	Female	25	105	136.31	126.10	Unknown cause	No	Yes	17.4	1.5	-
5	Female	28	66	113.07	176.16	DN	Yes	Yes	16.5	2.0	-
6	Female	30	81	92.60	188.33	Unknown cause	No	No	18.6	1.5	0.55 (low-average)
7	Female	33	109	50.72	168.62	Unknown cause	No	Yes	17.8	1.5	0.69 (high-average)
8	Female	39	68	72.23	178.58	Unknown cause	No	Yes	19.9	1.5	0.67 (high-average)
9	Female	38	58	71.04	250.38	LN	No	Yes	21.0	1.5	0.58 (low-average)
10	Female	38	71	54.29	112.59	IgA N	No	Yes	21.5	2.0	-
11	Female	46	69	18.89	114.51	Unknown cause	No	Yes	19.7	1.5	-
12	Female	45	68	149.58	242.33	Hypertention	No	Yes	17.6	1.5	-
13	Female	43	137	722.44	294.82	Hypertention	No	Yes	24.6	1.5	0.81 (high-average)
14	Female	50	67	128.91	180.66	IgA N	No	Yes	20.7	1.5	0.80 (high-average)
15	Female	50	84	12.03	147.08	Unknown cause	No	Yes	20.8	1.5	0.81 (high-average)
16	Female	50	79	133.98	80.81	FSGS	No	Yes	17.1	2.5	-
17	Female	49	62	71.70	121.25	Unknown cause	No	No	19.6	1.5	0.59 (low-average)

No.	Gender	Age (years)	PD vintage (months)	Dialysate MMP-7 (pg/ml)	Dialysate TGF-β1 (pg/ml)	Causes of ESRD	Diabetes mellitus	HBP	BMI (kg/m^2)	Glucose concentration (%)	PET
18	Female	54	50	76.63	193.25	Unknown cause	No	Yes	21.4	1.5	-
19	Female	54	56	43.98	104.95	Unknown cause	No	Yes	24.4	2.0	0.65 (low-average)
20	Female	55	53	38.64	131.62	Unknown cause	No	Yes	19.9	1.5	-
21	Female	51	67	94.42	63.54	Unknown cause	No	Yes	22.1	1.5	0.93 (high)
22	Female	58	103	88.52	154.09	Unknown cause	No	Yes	23.0	1.5	-
23	Female	54	128	310.08	126.42	Unknown cause	No	No	20.7	1.5	0.67 (high-average)
24	Men	29	52	123.82	136.86	Unknown cause	No	Yes	22.9	1.5	-
25	Men	35	71	131.84	107.17	Unknown cause	No	Yes	27.1	1.5	0.52 (low-average)
26	Men	32	152	82.52	212.23	Unknown cause	No	Yes	25.7	1.5	-
27	Men	32	94	175.72	301.51	Unknown cause	No	Yes	20.4	1.5	0.74 (high-average)
28	Men	33	146	196.98	162.16	FSGS	No	Yes	19.5	1.5	0.72 (high-average)
29	Men	35	104	148.10	286.15	IgA N	No	Yes	18.3	1.5	0.66 (high-average)
30	Men	38	103	72.76	89.53	Unknown cause	No	Yes	20.6	2.0	-
31	Men	35	120	94.14	182.05	Unknown cause	No	Yes	21.7	2.0	0.56 (low-average)
32	Men	36	66	68.67	295.65	RPGN	No	Yes	25.1	1.5	0.57 (low-average)
33	Men	37	83	168.97	136.20	Hypertention	No	Yes	22.8	1.5	0.78 (high-average)
34	Men	41	53	72.01	87.97	IgA N	No	Yes	23.4	2.0	0.86 (high)
35	Men	42	78	344.49	128.05	IgA N	No	No	19.9	1.5	0.61 (low-average)

No.	Gender	Age (years)	PD vintage (months)	Dialysate MMP-7 (pg/ml)	Dialysate TGF-β1 (pg/ml)	Causes of ESRD	Diabetes mellitus	HBP	BMI (kg/m^2)	Glucose concentration (%)	PET
36	Men	44	128	37.36	156.43	IgA N	No	No	32.0	2.5	1.01 (high)
37	Men	41	72	105.43	158.45	Hypertention	No	Yes	25.7	1.5	0.71 (high-average)
38	Men	45	76	83.41	371.41	Unknown cause	No	Yes	22.9	1.5	0.72 (high-average)
39	Men	43	89	297.44	227.29	MN	No	Yes	23.0	1.5	0.50 (low-average)
40	Men	46	105	73.29	176.51	IgA N	No	Yes	20.1	1.5	0.40 (low)
41	Men	46	52	52.38	245.00	Unknown cause	No	Yes	21.2	2.5	0.92 (high)
42	Men	46	88	116.93	136.20	Unknown cause	No	Yes	18.3	1.5	-
43	Men	48	130	163.11	575.55	Unknown cause	No	No	26.5	1.5	0.58 (low-average)
44	Men	50	92	320.21	198.92	Unknown cause	Yes	Yes	33.8	1.5	0.77 (high-average)
45	Men	48	108	96.13	179.27	Unknown cause	No	Yes	23.5	1.5	0.80 (high-average)
46	Men	49	111	24.16	115.47	Unknown cause	No	Yes	22.5	2.0	0.65 (low-average)
47	Men	52	110	555.15	330.45	Unknown cause	Yes	Yes	22.6	2.5	0.87 (high)
48	Men	53	45	66.57	120.61	Hypertention	No	Yes	24.9	1.5	0.70 (high-average)
49	Men	51	78	212.11	137.18	Unknown cause	No	Yes	30.4	1.5	-
50	Men	53	52	134.15	261.67	Unknown cause	No	No	26.7	2.0	0.69 (high-average)
51	Men	52	46	97.64	52.24	Unknown cause	No	No	22.4	2.5	0.73 (high-average)

The information of patients who provided dialysate effluents used in Fig. 2j, k. "-" indicates not available, *DN* diabetic nephropathy, *HBP* high blood pressure, *MN* membranous nephropathy, *LN* lupus nephritis, *FSGS* focal segmental glomerulosclerosis, *IgA N* IgA nephropathy, *RPGN* rapidly progressive glomerulonephritis, *PD* peritoneal dialysis, *MMP-7* matrix metalloproteinase-7, *TGF-β1* transforming growth factor-β1, *BMI* body mass index, *PET* peritoneal equilibration test, *ERSD* end-stage renal disease

Table S4 Genesets of fetal mesothelial cells hallmark, MMT, senescence, profibrosis, and ECM

Fetal mesothelial cells hallmark	MMT	Senescence	Profibrosis	ECM
ADAMTSL4-AS1, ALOX15, BDKRB1, C21orf62, CA11, EGOT, FENDRR, FGF9, GADL1, GFPT2, GLP2R, HAS1, HDAC1P1, KLK10, KRTDAP, LAMC2, LINC02364, MAP3K8, MEGF6, MIR548XHG, NFATC2, NSA2P7 PDZRN4, PLCB1, PODN, PSAPL1, RN7SL101P, RND1, RNU4-45P, RPL31P52, RPRD1B, SFTPD, SFTPD-AS1, TSHZ3-AS1	*ABI3BP, ACTA2, ADAM12, ANPEP, APLP1, AREG, BASP1, BDNF, BGN, BMP1, CADM1, CALD1, CALU, CAP2, CAPG, CCN1, CCN2, CD44, CD59, CDH11, CDH2, CDH6, COL11A1, COL12A1, COL16A1, COL1A1, COL1A2, COL3A1, COL4A1, COL4A2, COL5A1, COL5A2, COL5A3, COL6A2, COL6A3, COL7A1, COL8A2, COLGALT1, COMP, COPA, CRLF1, CTHRC1, CXCL1, CXCL12, CXCL6, CXCL8, DAB2, DCN, DKK1, DPYSL3, DST, ECM1, ECM2, EDIL3, EFEMP2, ELN, EMP3, ENO2, FAP, FAS, FBLN1, FBLN2, FBLN5, FBN1, FBN2, FERMT2, FGF2, FLNA, FMOD, FN1, FOXC2, FSTL1, FSTL3, FUCA1, FZD8, GADD45A, GADD45B, GAS1, GEM, GJA1,GLIPR1 GPC1, GPX7, GREM1 HTRA1, ID2, IGFBP2, IGFBP3, IGFBP4, IL15, IL32, IL6, INHBA, ITGA2, ITGA5, ITGAV, ITGB1, ITGB3, ITGB5, JUN, LAMA1, LAMA2, LAMA3, LAMC1, LAMC2, LGALS1, LOX, LOXL1, LOXL2, LRP1, LRRC15, LUM, MAGEE1, MATN2, MATN3, MCM7, MEST, MFAP5, MGP, MMP1, MMP14,*	*ALDH18A1, CDKN2A, CDKN2B, ERGIC1, VDAC3, FLII, TOMM70A, VDAC1, GDF15, GPX1, IDH2, ATL3, SPON1, OSTF1, ARPC5L, ATP6V0A1, RHOC, CLU, VDAC2, LARS, ACADM, SNRPB, SNRPN, TNFAIP8, HBA1, CKM, HMGA1, TGM2, TUBB1, TUBB2A, EEF1A1, XPNPEP3, TUBA1A, TUBB4B, GALE, CANX, PKM, USP9X, RPS11, TUBB6, IGFBP2, CYB5R3, ASPH, TUBA4B, GYS1, SLC25A24, DYNC1H1, TMEM109, IQGAP1, FUCA1, LRBA, CXCL8, LAMA3, SCARB2, TUBB, TUBA8, CKB, CAPN2, TUBAL3, TUBB2B, ARL6IP5, PRDX5, EEF1E1, SLC25A6, PFKP, DDOST, VARS, ATP5O, HSD17B10, HBD, CSRP2, CRYAB, CRYM, CMBL,CLIC1, ELAVL1, RHOA, EPS8L2, S100A10, TUBB8, ATP6V1E1, MYLK3, TFPI2, ANXA1, NOL3, HSPA9, GAPDH, MAPK13, FAM129B, HADHA, APRT, TUBB3, EIF4A2, ANXA2, LAMTOR1, PDLIM1, ACTB, SOD2, GALNS, RPS20, VASP, CES2, TMED9, COX5B, RAB10, AK1, IMPDH2, DYNLRB1, KIF5B, NCK2, S100A16, CPNE1, RPL11, ME2, VAT1, STRBP, TUFM, USO1, VTN, SNX2, HTRA1, DDRGK1, C21ORF33, RAB15, CORO1B, ILK, PGM2L1, TUBA4A, RAN, CRIP2, PSMC2, LRRC47, TUBB4A, TES, ACTC1, SNRPE, CAPNS1, TMED10, MYLK, FLOT1, MYOF, RAB6A, PSMD7, ACTBL2, STC2, TPD52L2, SDHB, PDLIM4, UBE2V1, DPYSL2, PPP1R7, UBA1, HSPA1A, FBL, MAP4, H2AFY, RPS3A, PHB, PGRMC1, YBX1, TLN1, RPS4X, SQRDL, IMMT, PFN2, PAFAH1B2, BDH2, UGDH, RPL14, SFPQ, SEC31A, CLTC, RPL4, RPL18A, ATP5A1, FLOT2, CSRP1, MYL12B, FAM129A, RAB33B, PLEC, S100A11, MPST, GSTO1, GUK1, DSTN, ARPC1B, RPL5, POTEF, GAA, POLR3C, UGP2, SEC22B, PICALM, MACF1, DLST, PPP1CA, TUBA1C, DCN, GLRX, CBR1, RPL7A, PTGR1, KRT86, CAPG, POTEJ, PTER, NCSTN, COPG1, SCIN, HIST2H3A, APP, TINAGL1, APLP2, GPI, TPM1, ICOSLG, SRSF2, LDHB, CXCL1, SOD1, PSMB4, TGFBI, PTGFRN, APEX1, CD81, DBI,CYCS, SUMO1, HYPK,*	*PDGFB, TGFB1, COL3A1, ACTA2, MMP2, PDGFA, VIM, NKD2, TNC, GPNMB, CD9, TNFSF12, TL2R, TNF, IGF1, TIMP2, ANXA5, FN1, SPP1, ECM1, MMP12, MMP14, MMP9, MMP19, CTSB, CTSD, CTSZ, CTSL, CTSS, ARG1, PF4,*	*FN1, SPP1, ECM1, MMP12, MMP14, MMP9, MMP19, CTSB, CTSD, CTSZ, CTSL, CTSS, ARG1, PF4, THBS1, IGFBP1, IGFBP2, IGFBP3, IGFBP4, IGFBP5, IGFBP6, IGFBP7, COL1A1, COL1A2, COL2A1, COL3A1, COL4A1,*

Fetal mesothelial cells hallmark	MMT	Senescence	Profibrosis	ECM
	MMP2, MMP3, MSX1, MXRA5, MYL9, MYLK, NID2, NNMT, NOTCH2, NT5E, NTM, OXTR, P3H1, PCOLCE, PCOLCE2, PDGFRB, PDLIM4, PFN2, PLAUR, PLOD1, PLOD2, PLOD3, PMEPA1, PMP22, POSTN, PPIB, PRRX1, PRSS2, PTHLH, PTX3, PVR, QSOX1, RGS4, RHOB, SAT1, SCG2, SDC1, SDC4, SERPINE1, SERPINE2, SERPINH1, SFRP1, SFRP4, SGCB, SGCD, SGCG, SLC6A8, SLIT2, SLIT3, SNAI2, SNTB1, SPARC, SPOCK1, SPP1, TAGLN, TFPI2, TGFB1, TGFBI, TGFBR3, TGM2, THBS1, THBS2, THY1, TIMP1, TIMP3, TNC, TNFAIP3, TNFRSF11B, TNFRSF12A, TPM1, TPM2, TPM4, VCAM1, VCAN, VEGFA, VEGFC, VIM, WIPF1, WNT5A	*SDCBP*, *SRSF7*, *B4GALT1*, *SERPINE2*, *LDLR*, *IGFBP6*, *NRP1*, *DPY30*, *TPM2*, *LTBP2*, *GFRA1*, *KRT10*, *CALR*, *TIMP2*, *RPL10A*, *PRNP*, *FABP5*, *ANP32B*, *MAN1A1*, *LMNB1*, *BHMT*, *TPT1*, *SERPING1*, *ANP32A*, *ECM1*, *NEO1*, *ATP6AP1*, *PCNA*, *SRSF1*, *MYL9*, *PROS1*, *GALNT7*, *FBN1*, *DTD1*, *STMN1*, *ERH*, *PFKL*, *HN1L*, *CTSZ*, *FBLN1*, *CTGF*, *UHRF2*, *PTK7*, *IGFBP7*, *SIRPA*, *SIRPB1*, *ATRN*, *AGRN*, *NPC2*, *LRRN4*, *CFI*, *CFB*, *CBX3*, *BLMH*, *VASN*, *SMAP*, *ADAM10*, *HIST1H2AG*, *THBS1*, *GALNT2*, *DCTPP1*, *COL7A1*, *EPHB2*, *C1S*, *COL6A1*, *VCAM1*, *FDPS*, *THBS2*, *CDH6*, *SET*, *PVR*, *SOD3*, *LGALS3BP*, *TXNDC12*, *AP2A2*, *TWSG1*, *HIST1H1E*, *RPS27A*, *F5*, *CFDP1*, *DAG1*, *SERPINF1*, *DNAJC8*, *SPOCK1*, *GOLM1*, *NID1*, *PTPRK*, *COL4A2*, *TAGLN*, *LUM*, *HLA-C*, *CBX5*, *VCAN*, *NASP*, *LOXL2*, *GC*, *COL1A1*, *COL4A1*, *PROCR*, *PCBD1*, *FN1*, *MYO1F*, *NLN*, *FSTL1*, *TIMP1*, *IGFBP4*, *B2M*, *SERPINE1*, *A2M*, *PTGDS*, *ALB*, *AEBP1*, *MMP9*, *FXYD2*, *SPARC*, *TF*, *LTF*, *AXL*, *RARRES2*, *PZP*, *SERPINA1*, *HBE1*, *HBB*, *HBG1*, *HBG2*, *MYLK4*, *MYLK2*, *MAPK8*, *MAPK9*, *MAPK12*, *MAPK10*, *MAPK11*, *MAPK14*, *ACTG1*, *NCK1*, *HIST1H1D*, *HIST1H1C*, *HIST1H1T*, *HIST1H1A*, *HIST1H2AD*, *HIST1H2AH*, *HIST1H2AJ*, *H2AFJ*, *KRT81*, *KRT83*, *CXCL3*, *CXCL2*, *RAB6B*, *HSPA1B*, *MYL12A*	*THBS1*	*COL4A2*, *COL4A3*, *COL4A4*, *COL4A5*, *COL4A6*, *COL5A1*

MMT mesothelial-mesenchymal transition, *ECM* extracellular matrix, *ADAMTSL4-AS1* ADAMTSL4 antisense RNA 1, *ALOX15* arachidonate 15-lipoxygenase, *BDKRB1* bradykinin receptor B1, *CA11* carbonic anhydrase 11, *EGOT* eosinophil granule ontogeny transcript, *FENDRR* FOXF1 adjacent non-coding developmental regulatory RNA, *FGF9* fibroblast growth factor 9, *GADL1* glutamate decarboxylase like 1, *GFPT2* glutamine-fructose-6-phosphate transaminase 2, *GLP2R* glucagon like peptide 2 receptor, *HAS1* hyaluronan synthase 1, *HDAC1P1* histone deacetylase 1 pseudogene 1, *KLK10* kallikrein related peptidase 10, *KRTDAP k*eratinocyte differentiation associated protein, *LAMC2* laminin subunit gamma 2, *LINC02364* long intergenic non-protein coding RNA 2364, *MAP3K8* mitogen-activated protein kinase kinase kinase 8, *MEGF6* multiple EGF like domains 6, *MIR548XHG* MIR548X host gene, *NFATC2* nuclear factor of activated T cells 2, *NSA2P7* NSA2 pseudogene 7, *PDZRN4 PDZ* domain containing ring finger 4, *PLCB1* phospholipase C beta 1, *PODN* podocan, *PSAPL1* prosaposin like 1, *RN7SL101P* RNA 7SL cytoplasmic 101 pseudogene, *RND1* Rho family GTPase 1, *RNU4-45P* RNA U4 small nuclear 45 pseudogene, *RPL31P52* ribosomal protein L31 pseudogene 52, *RPRD1B* regulation of nuclear pre-mRNA domain containing 1B, *SFTPD* surfactant protein D, *SFTPD-AS1* SFTPD antisense RNA 1, *TSHZ3-AS1* TSHZ3 antisense RNA 1, *ABI3BP* target of Nesh-SH3, *ACTA2* actin, aortic smooth muscle, *ADAM12* disintegrin and metalloproteinase domain-containing protein 12, *ANPEP* aminopeptidase N, *APLP1* amyloid beta precursor like protein 1, *AREG* amphiregulin, *BASP1* brain

acid soluble protein 1, *BDNF* neurotrophic factor BDNF precursor form, *BGN* biglycan, *BMP1* bone morphogenetic protein 1, *CADM1* cell adhesion molecule 1, *CALD1* Caldesmon 1, *CALU* calumenin, *CAP2* adenylyl cyclase-associated protein 2, *CAPG* macrophage-capping protein, *CCN1* cellular communication network factor 1, *CCN2* cellular communication network factor 2, *CDH11* cadherin-11, *CDH2* cadherin-2, *CDH6* cadherin 6, *COL11A1* collagen type XI alpha 1 chain, *COL12A1* collagen type XII alpha 1 chain, *COL16A1* collagen type XVI alpha 1 chain, *COL1A1* collagen type I alpha 1 chain, *COL1A2* collagen type I alpha 2 isoform 1, *COL3A1* collagen type III alpha 1 chain, *COL4A1* collagen type IV alpha 1 chain, *COL4A2* collagen type IV alpha 2 chain, *COL5A2* collagen type V alpha 2 chain, *COL5A3* collagen type V alpha 3 chain, *COL6A2* collagen type VI alpha 2 chain, *COL6A3* collagen type VI alpha 3 chain, *COL7A1* collagen type VII alpha 1, *COL8A2* collagen type VIII alpha 2, *COLGALT1* procollagen galactosyltransferase 1, *COMP* cartilage oligomeric matrix protein, *COPA* coatomer subunit alpha, *CRLF1* cytokine receptor-like factor 1, *CTHRC1* collagen triple helix repeat-containing protein 1, *CXCL1* C-X-C motif chemokine ligand 1, *CXCL12* C-X-C motif chemokine ligand 12, *CXCL6* C-X-C motif chemokine 6, *CXCL8* C-X-C motif chemokine ligand 8, *DAB2* disabled homolog 2, *DCN* decorin, *DKK1* Dickkopf-like protein 1, *DPYSL3* dihydropyrimidinase-related protein 3, *DST* dystonin, *ECM1* extracellular matrix protein 1, *ECM2* extracellular matrix protein 2, *EDIL3* EGF-like repeat and discoidin I-like domain-containing protein 3, *EFEMP2* EGF-containing fibulin-like extracellular matrix protein 2, *ELN* elastin, *EMP3* epithelial membrane protein 3, *ENO2* enolase 2, *FAP* prolyl endopeptidase FAP, *FAS* fatty acid synthase, *FBLN1* fibulin 1, *FBLN2* fibulin 2, *FBLN5* fibulin 5, *FBN1* fibrillin 1, *FBN2* fibrillin 2, *FERMT2* fermitin family homolog 2, *FGF2* fibroblast growth factor, *FLNA* filamin A, *FMOD* fibromodulin, *FN1* fibronectin, *FOXC2* forkhead box protein C2, *FSTL1* follistatin-related protein 1, *FSTL3* follistatin-related protein 3, *FUCA1* alpha-L-fucosidase, *FZD8* frizzled-8, *GADD45A* growth arrest and DNA damage-inducible protein GADD45 alpha, *GADD45B* growth arrest and DNA damage-inducible protein GADD45 beta, *GAS1* growth arrest-specific protein 1, *GEM* GTP-binding protein GEM, *GJA1* gap junction alpha-1 protein, *GLIPR1* glioma pathogenesis-related protein 1, *GPC1* glypican-1, *GPX7* glutathione peroxidase 7, *GREM1* gremlin 1, *HTRA1* HtrA serine peptidase 1, *ID2* DNA-binding protein inhibitor ID-2, *IGFBP2* insulin-like growth factor-binding protein 2, *IGFBP3* insulin-like growth factor-binding protein 3, *IGFBP4* insulin-like growth factor-binding protein 4, *IL15* interleukin-15, *IL32* interleukin-32, *IL6* interleukin-6, *INHBA* inhibin beta A chain, *ITGA2* integrin alpha-2, *ITGA5* integrin alpha-5, *ITGAV* integrin alpha-V, *ITGB1* integrin beta-1, *ITGB3* integrin subunit beta 3, *ITGB5* integrin subunit beta 5, *JUN* transcription factor Jun, *LAMA1* laminin subunit alpha-1, *LAMA2* laminin subunit alpha-2, *LAMA3* laminin subunit alpha-3, *LAMC1* laminin subunit gamma-1, *LAMC2* laminin subunit gamma-2, *LGALS1* galectin-1, *LOX* lysyl oxidase, *LOXL1* lysyl oxidase homolog 1, *LOXL2* lysyl oxidase homolog 2, *LRP1* prolow-density lipoprotein receptor-related protein 1, *LRRC15* leucine-rich repeat-containing protein 15, *LUM* lumican, *MAGEE1* melanoma-associated antigen E1, *MATN2* matrilin 2, *MATN3* matrilin-3, *MCM7* minichromosome maintenance complex component 7, *MEST* mesoderm specific transcript, *MFAP5* microfibrillar-associated protein 5, *MGP* matrix Gla protein, *MMP1* matrix metalloproteinase-1, *MMP14* matrix metalloproteinase-14, *MMP2* matrix metalloproteinase-2, *MMP3* matrix metalloproteinase-3, *MSX1* Msh homeobox 1, *MXRA5* matrix-remodeling-associated protein 5, *MYL9* myosin regulatory light polypeptide 9, *MYLK* myosin light chain kinase, *NID2* nidogen-2, *NNMT* nicotinamide N-methyltransferase, *NOTCH2* notch receptor 2, *NT5E* 5'-nucleotidase, *NTM* neurotrimin, *OXTR* oxytocin receptor, P3H1 prolyl 3-hydroxylase 1, *PCOLCE* procollagen C-endopeptidase enhancer 1, *PCOLCE2* procollagen C-endopeptidase enhancer 2, *PDGFRB* platelet-derived growth factor receptor beta, *PDLIM4* PDZ and LIM domain protein 4, *PFN2* profilin-2, *PLAUR* urokinase plasminogen activator surface receptor, *PLOD1* procollagen-lysine,2-oxoglutarate 5-dioxygenase 1, *PLOD2* procollagen-lysine, 2-oxoglutarate 5-dioxygenase 2, *PLOD3* procollagen-lysine, 2-oxoglutarate 5-dioxygenase 3, *PMEPA1* prostate transmembrane protein androgen induced 1, *PMP22* peripheral myelin protein 22, *POSTN* periostin, *PPIB* peptidyl-prolyl cis-trans isomerase B, *PRRX1* paired mesoderm homeobox protein 1, *PRSS2* serine protease 2, *PTHLH* parathyroid hormone-related protein, *PTX3* pentraxin-related protein 3, *PVR* poliovirus receptor, *QSOX1* quiescin sulfhydryl oxidase 1, *RGS4* regulator of G-protein signaling 4, *RHOB* ras homolog family member B, *SAT1* spermidine/spermine N1-acetyltransferase 1, *SCG2* secretogranin-2, *SDC1* syndecan-1, *SDC4* syndecan-4, *SERPINE1* serpin family E

member 1, *SFRP1* secreted frizzled-related protein 1, *SFRP4* secreted frizzled-related protein 4, *SGCB* beta-sarcoglycan, *SGCD* delta-sarcoglycan, *SGCG* gamma-sarcoglycan, *SLC6A8* sodium- and chloride-dependent creatine transporter 1, *SLIT2* slit guidance ligand 2, *SLIT3* slit guidance ligand 3, *SNAI2* snail family transcriptional repressor 2, *SNTB1* beta-1-syntrophin, *SPARC* secreted protein acidic and cysteine rich, *SPOCK1* SPARC (osteonectin), cwcv and kazal like domains proteoglycan 1, *SPP1* secreted phosphoprotein 1, *TAGLN* transgelin, *TFPI2* tissue factor pathway inhibitor 2, *TGFB1* transforming growth factor beta-1, *TGFBI* transforming growth factor-beta-induced, *TGFBR3* transforming growth factor beta receptor type 3, *TGM2* transglutaminase 2, *THBS1* thrombospondin-1, *THBS2* thrombospondin-2, *THY1* Thy-1 cell surface antigen, *TIMP1* TIMP metallopeptidase inhibitor 1, *TIMP3* TIMP metalloproteinase inhibitor 3, *TNC* tenascin, *TNFAIP3* TNF alpha induced protein 3, *TNFRSF11B* tumor necrosis factor receptor superfamily member 11B, *TNFRSF12A* tumor necrosis factor receptor superfamily member 12A, *TPM1* tropomyosin-1, *TPM4* tropomyosin-4, *VCAM1* vascular cell adhesion protein 1, *VCAN* versican, *VEGFA* vascular endothelial growth factor A, *VEGFC* vascular endothelial growth factor C, *VIM* vimentin, *WIPF1* WAS/WASL-interacting protein family member 1, *WNT5A* Wnt family member 5A. *ALDH18A1* aldehyde dehydrogenase 18 family member A1, *CDKN2A* cyclin-dependent kinase inhibitor 2A, *CDKN2B* cyclin-dependent kinase 4 inhibitor B, *ERGIC1* endoplasmic reticulum-Golgi intermediate compartment protein 1, *VDAC3* voltage dependent anion channel 3, *FLII* actin remodeling protein, *TOMM70A* mitochondrial import receptor subunit TOM70, *VDAC1* voltage-dependent anion-selective channel protein 1, *GDF15* growth/differentiation factor 15, *GPX1* glutathione peroxidase 1, *IDH2* isocitrate dehydrogenase ($NADP^+$) 2, *ATL3* atlastin-3, *SPON1* spondin-1, *OSTF1* osteoclast-stimulating factor 1, *ARPC5L* actin-related protein 2/3 complex subunit 5-like protein, *ATP6V0A1* ATPase H^+ transporting V0 subunit a1, *RHOC* ras homolog family member C, *CLU* clusterin, *VDAC2* voltage dependent anion channel 2, *LARS* leucine-tRNA ligase, *ACADM* acyl-CoA dehydrogenase medium chain, *SNRPB* small nuclear ribonucleoprotein-associated protein, *SNRPN* small nuclear ribonucleoprotein-associated protein N, *TNFAIP8* tumor necrosis factor alpha-induced protein 8, *HBA1* hemoglobin subunit alpha, *CKM* creatine kinase M-type, *HMGA1* high mobility group AT-hook 1, *TUBB1* tubulin beta 1 class VI, *TUBB2A* tubulin beta 2A class IIa, *EEF1A1* elongation factor 1-alpha 1, *XPNPEP3* X-prolyl aminopeptidase 3, *TUBA1A* tubulin alpha-1A chain, *TUBB4B* tubulin beta-4B chain, GALE UDP-glucose 4-epimerase, *CANX* calnexin, *PKM* pyruvate kinase, *USP9X* ubiquitinyl hydrolase 1, *RPS11* small ribosomal subunit protein uS17, *TUBB6* Tubulin beta 6 class V, *CYB5R3* NADH-cytochrome b5 reductase 3, *ASPH* Aspartyl/asparaginyl beta-hydroxylase, *TUBA4B* tubulin-like protein alpha-4B, *GYS1* glycogenin-1, *SLC25A24* solute carrier family 25 member 24, *DYNC1H1* dynein cytoplasmic 1 heavy chain 1, *TMEM109* transmembrane protein 109, *IQGAP1* IQ motif containing GTPase activating protein 1, *LRBA* lipopolysaccharide-responsive and beige-like anchor protein, *SCARB2* scavenger receptor class B member 2, *TUBB* tubulin beta chain, *TUBA8* tubulin alpha-8 chain, CKB creatine kinase B-type, *CAPN2* calpain-2 catalytic subunit, *TUBAL3* tubulin alpha chain-like 3, *TUBB2B* tubulin beta-2B chain, *ARL6IP5* ARF like GTPase 6 interacting protein 5, *PRDX5* peroxiredoxin-5, *EEF1E1* eukaryotic translation elongation factor 1 epsilon-1, *SLC25A6* solute carrier family 25 member 6, *PFKP* phosphofructokinase, platelet, *DDOST* dolichyl-diphosphooligosaccharide-protein glycosyltransferase non-catalytic subunit, *VARS* valine-tRNA ligase, *ATP5O* ATP synthase peripheral stalk subunit OSCP, *HSD17B10* 3-hydroxyacyl-CoA dehydrogenase type-2, *HBD* hemoglobin subunit delta, *CSRP2* cysteine and glycine-rich protein 1, *CRYAB* crystallin B chain, *CRYM* crystallin mu, *CMBL* carboxymethylenebutenolidase homolog, *CLIC1* chloride intracellular channel protein, *ELAVL1* ELAV-like protein 1, *RHOA* ras homolog family member A, *EPS8L2* epidermal growth factor receptor kinase substrate 8-like protein 2, *S100A10* S100 calcium binding protein A10 *TUBB8* tubulin beta 8 class VII, *ATP6V1E1* V-type proton ATPase subunit E 1, *MYLK3* myosin light chain kinase 3, *ANXA1* annexin A1, *NOL3* nucleolar protein 3, *HSPA9* heat shock protein family A (Hsp70) member 9, *GAPDH* glyceraldehyde-3-phosphate dehydrogenase (phosphorylating), *MAPK13* mitogen-activated protein kinase 13, *HADHA* hydroxyacyl-CoA dehydrogenase trifunctional multienzyme complex subunit alpha, *APRT* adenine phosphoribosyltransferase, *TUBB3* tubulin beta-3 chain, *EIF4A2* eukaryotic translation initiation factor 4A2, *ANXA2* annexin A2, *LAMTOR1* late endosomal/lysosomal adaptor, MAPK and MTOR activator 1, *PDLIM1* PDZ and LIM domain protein 1, *ACTB* actin beta, *SOD2* superoxide

dismutase 2, *GALNS* galactosamine (N-acetyl)-6-sulfatase, *RPS20* ribosomal protein S20, *VASP* vasodilator-stimulated phosphoprotein, *CES2* cocaine esterase, *TMED9* transmembrane emp24 domain-containing protein 9, *COX5B* cytochrome c oxidase subunit 5B, *RAB10* RAB10, member RAS oncogene family, *AK1* adenylate kinase isoenzyme 1, *IMPDH2* inosine-5'-monophosphate dehydrogenase 2, *DYNLRB1* dynein light chain roadblock-type 1, *KIF5B* kinesin family member 5B, *NCK2* NCK adaptor protein 2, *S100A16* S100 calcium binding protein A16, *CPNE1* copine-1, *RPL11* ribosomal protein L11, *ME2* malic enzyme 2, *VAT1* vesicle amine transport 1, *STRBP* spermatid perinuclear RNA-binding protein, *TUFM* elongation factor Tu, *USO1* general vesicular transport factor p115, *VTN* vitronectin, *SNX2* sorting nexin-2, *DDRGK1* DDRGK domain-containing protein 1, *C21ORF33* glutamine amidotransferase-like class 1 domain-containing protein 3, *CORO1B* coronin-1B, ILK integrin linked kinase, *PGM2L1* phosphoglucomutase 2 like 1, *TUBA4A* tubulin alpha 4a, *RAN* GTP-binding nuclear protein Ran, *CRIP2* cysteine-rich protein 2, *PSMC2* 26S proteasome regulatory subunit 7, *LRRC47* leucine-rich repeat-containing protein 47, *TUBB4A* tubulin beta 4A class IVa, *TES* testin, *ACTC1* actin alpha cardiac muscle 1, *SNRPE* small nuclear ribonucleoprotein E, *CAPNS1* calpain small subunit 1, *TMED10* transmembrane emp24 domain-containing protein 10, *FLOT1* flotillin-1, *MYOF* myoferlin, *RAB6A* Ras-related protein Rab-6A, *PSMD7* 26S proteasome non-ATPase regulatory subunit 7, *ACTBL2* beta-actin-like protein 2, *STC2* stanniocalcin-2, *TPD52L2* tumor protein D52-like 2, *SDHB* succinate dehydrogenase complex iron sulfur subunit B, *UBE2V1* ubiquitin-conjugating enzyme E2 variant 1, *DPYSL2* dihydropyrimidinase-related protein 2, *PPP1R7* protein phosphatase 1 regulatory subunit 7, *UBA1* ubiquitin-like modifier-activating enzyme 1, *HSPA1A* heat shock protein family A (Hsp70) member 1A, *FBL* fibrillarin, *MAP4* microtubule-associated protein 4, *RPS3A* ribosomal protein S3A, *PHB* prohibitin 1, *PGRMC1* progesterone receptor membrane component 1, *YBX1* Y-box-binding protein 1, *TLN1* talin-1, *RPS4X* 40S ribosomal protein S4, *SQRDL* sulfide quinone oxidoreductase, *IMMT* inner membrane mitochondrial protein, *PAFAH1B2* platelet-activating factor acetylhydrolase IB subunit alpha2, *BDH2* 3-hydroxybutyrate dehydrogenase 2, *UGDH* UDP-glucose 6-dehydrogenase, *RPL14* ribosomal protein L14, *SFPQ* splicing factor proline and glutamine rich, *SEC31A* Protein transport protein Sec31A, *CLTC* clathrin heavy chain 1, *RPL4* ribosomal protein L4, *RPL18A* ribosomal protein L18a, *ATP5A1* ATP synthase F1 subunit alpha, *FLOT2* flotillin 2, *CSRP1* cysteine and glycine-rich protein 1, *MYL12B* myosin regulatory light chain 12B, *RAB33B* Ras-related protein Rab-33B, *PLEC* plectin, *S100A11* S100 calcium binding protein A11, *MPST* 3-mercaptopyruvate sulfurtransferase, *GSTO1* glutathione S-transferase omega-1, *GUK1* Guanylate kinase 1, *DSTN* destrin, *ARPC1B* actin-related protein 2/3 complex subunit 1B, *RPL5* ribosomal protein L5, *POTEF* POTE ankyrin domain family member F, *GAA* alpha-glucosidase, *POLR3C* RNA polymerase III subunit C, *UGP2* UTP-glucose-1-phosphate uridylyltransferase, *SEC22B* vesicle-trafficking protein SEC22b, *PICALM* phosphatidylinositol-binding clathrin assembly protein, *MACF1* microtubule actin crosslinking factor 1, *DLST* dihydrolipoamide S-succinyltransferase, *PPP1CA* protein phosphatase 1, *TUBA1C* tubulin alpha-1C chain, *GLRX* glutaredoxin-1, CBR1 carbonyl reductase 1, *RPL7A* ribosomal protein L7A, *PTGR1* Prostaglandin reductase 1, *KRT86* keratin 86, *POTEJ* POTE ankyrin domain family member J, *PTER* phosphotriesterase related, *COPG1* coatomer subunit gamma-1, *SCIN* scinderin, *HIST2H3A* histone H3.2, *APP* amyloid beta precursor protein, *TINAGL1* tubulointerstitial nephritis antigen-like, *APLP2* amyloid beta precursor like protein 2, *GPI* glucose-6-phosphate isomerase, *ICOSLG* inducible T cell costimulator ligand *LDHB* L-lactate dehydrogenase B chain, *SOD1* superoxide dismutase 1, *PSMB4* proteasome subunit beta type-4, *PTGFRN* prostaglandin F2 receptor negative regulator, *APEX1* apurinic/apyrimidinic endodeoxyribonuclease 1, *CD81* CD81 antigen, *DBI* diazepam binding inhibitor, *CYCS* cytochrome C, *SUMO1* small ubiquitin-related modifier 1, *HYPK* huntingtin-interacting protein K, *SDCBP* syntenin-1, *SRSF7* serine/arginine-rich splicing factor 7, *B4GALT1* beta-1,4-galactosyltransferase 1, *SERPINE2* serpin family E member 2, *LDLR* low-density lipoprotein receptor, *IGFBP6* insulin-like growth factor-binding protein 6, *NRP1* neuropilin-1, *DPY30* dpy-30 histone methyltransferase complex regulatory subunit, *TPM2* tropomyosin-2, *LTBP2* latent-transforming growth factor beta-binding protein 2, *GFRA1* GDNF family receptor alpha-1, *KRT10* keratin 10, *CALR* calreticulin, *TIMP2* TIMP metalloproteinase inhibitor 2, *RPL10A* ribosomal protein L10a, *PRNP* prion protein, *FABP5* fatty acid-binding protein 5, *ANP32B* acidic leucine-rich nuclear phosphoprotein 32 family member B, *MAN1A1* mannosyl-oligosaccharide 1,2-alpha-mannosidase IA,

LMNB1 lamin-B1, *BHMT* betaine-homocysteine S-methyltransferase 1, *TPT1* translationally-controlled tumor protein, *SERPING1* serpin family G member 1, *ANP32A* acidic leucine-rich nuclear phosphoprotein 32 family member, *NEO1* neogenin, *ATP6AP1* V-type proton ATPase subunit S1, *PCNA* proliferating cell nuclear antigen, *SRSF1* serine/arginine-rich splicing factor 1, *PROS1* protein S, *GALNT7* polypeptide N-acetylgalactosaminyltransferase 7, *DTD1* D-aminoacyl-tRNA deacylase, *STMN1* stathmin, *ERH* enhancer of rudimentary homolog, *PFKL* phosphofructokinase, liver type, HN1L hematological and neurological expressed 1-like protein, *CTSZ* cathepsin Z, *CTGF* connective tissue growth factor, *UHRF2* ubiquitin like with PHD and ring finger domains 2, *PTK7* protein tyrosine kinase 7, *IGFBP7* insulin-like growth factor-binding protein 7, *SIRPA* signal regulatory protein alpha, *SIRPB1* signal-regulatory protein beta-1, *ATRN* attractin, *AGRN* agrin, *NPC2* NPC intracellular cholesterol transporter 2, *LRRN4* leucine rich repeat neuronal 4, *CFI* complement factor I, *CFB* complement factor B, *CBX3* chromobox 3, *BLMH* bleomycin hydrolase, *VASN* vasorin, *SMAP* skeletal muscle abundant protein, *ADAM10* ADAM metallopeptidase domain 10, *HIST1H2AG* histone H2A type 1-G, *GALNT2* polypeptide N-acetylgalactosaminyltransferase 2, *DCTPP1* dCTP pyrophosphatase 1, *EPHB2* ephrin type-B receptor 2, *C1S* complement C1s subcomponent, *COL6A1* collagen type VI alpha 1 chain, *FDPS* farnesyl diphosphate synthase, *SET* SET nuclear proto-oncogene, *SOD3* superoxide dismutase 3, *LGALS3BP* galectin-3-binding protein, *TXNDC12* thioredoxin domain-containing protein 12, *AP2A2* AP-2 complex subunit alpha-2, *TWSG1* twisted gastrulation protein homolog 1, *HIST1H1E* histone H1.4, *RPS27A* ubiquitin-ribosomal protein eS31 fusion protein, *F5* coagulation factor V, *CFDP1* craniofacial development protein 1, *DAG1* dystroglycan 1, *SERPINF1* serpin family F member 1, *DNAJC8* DnaJ homolog subfamily C member 8, *GOLM1* golgi membrane protein 1, *NID1* nidogen-1, *PTPRK* protein tyrosine phosphatase receptor type K, *HLA-C* HLA class I histocompatibility antigen, C alpha chain, *CBX5* chromobox protein homolog 5, *NASP* nuclear autoantigenic sperm protein, *GC* GC vtamin D-binding protein, *PROCR* protein C receptor, *PCBD1* pterin-4-alpha-carbinolamine dehydratase, *MYO1F* unconventional myosin-If, *NLN* neurolysin, *B2M* beta-2-microglobulin, *A2M* alpha-2-macroglobulin, *PTGDS* prostaglandin-H2 D-isomerase, *ALB* albumin, *AEBP1* adipocyte enhancer-binding protein 1, *MMP9* matrix metalloproteinase-9, *FXYD2* FXYD domain containing ion transport regulator 2, *TF* transferrin, *LTF* lactotransferrin, *AXL* AXL receptor tyrosine kinase, *RARRES2* retinoic acid receptor responder 2, *PZP* PZP alpha-2-macroglobulin like, *SERPINA1* serpin family A member 1, *HBE1* hemoglobin subunit epsilon, *HBB* hemoglobin subunit beta, *HBG1* hemoglobin subunit gamma-1, *HBG2* hemoglobin subunit gamma-2, *MYLK4* myosin light chain kinase family member 4, *MYLK2* myosin light chain kinase 2, *MAPK8* mitogen-activated protein kinase 8, *MAPK9* mitogen-activated protein kinase 9, *MAPK12* mitogen-activated protein kinase 12, *MAPK10* mitogen-activated protein kinase 10, *MAPK11* mitogen-activated protein kinase 11, *MAPK14* mitogen-activated protein kinase 14, *ACTG1* actin gamma 1, *NCK1* NCK adaptor protein 1, *HIST1H1D* histone H1.3, *HIST1H1C* histone H1.2, *HIST1H1T* histone H1t, *HIST1H1A* histone H1.1, *HIST1H2AD* histone H2A type 1-D, *HIST1H2AH* histone H2A type 1-H, *HIST1H2AJ* histone H2A type 1-J, *H2AFJ* histone H2A.J, *KRT81* keratin-81, *KRT83* keratin-83, *CXCL3* C-X-C motif chemokine 3, *CXCL2* C-X-C motif chemokine 2, *RAB6B* Ras-related protein Rab-6B, *HSPA1B* heat shock protein family A (Hsp70) member 1B, *MYL12A* myosin regulatory light chain 12A, *PDGFB* platelet-derived growth factor subunit B, *PDGFA* platelet-derived growth factor subunit A, *NKD2* protein naked cuticle homolog 2, *TNC* tenascin C, *GPNMB* transmembrane glycoprotein NMB, *CD9* CD9 antigen, *TNFSF12* tumor necrosis factor ligand superfamily member 12, *TNF* tumor necrosis factor, *IGF1* Insulin-like growth factor 1, *ANXA5* Annexin A5, *MMP12* matrix metalloproteinase-12, *CTSB* cathepsin B, *CTSD* cathepsin D, *CTSL* cathepsin L, *CTSS* cathepsin S *ARG1* arginase-1, *MMP19* matrix metalloproteinase-19, *PF4* platelet factor 4, *IGFBP1*insulin-like growth factor-binding protein 1, *IGFBP5* insulin like growth factor binding protein 5, *COL2A1* collagen alpha 1 type II, *COL4A3* collagen type IV alpha 3 chain, *COL4A4* collagen type IV alpha 4 chain, *COL4A5* collagen type IV alpha 5 chain, *COL4A6* collagen type IV alpha 6 chain, *COL5A1* collagen type V alpha 1 chain
